# Complete Mitochondrial Genomes of New Zealand’s First Dogs

**DOI:** 10.1371/journal.pone.0138536

**Published:** 2015-10-07

**Authors:** Karen Greig, James Boocock, Stefan Prost, K. Ann Horsburgh, Chris Jacomb, Richard Walter, Elizabeth Matisoo-Smith

**Affiliations:** 1 Allan Wilson Centre, Department of Anatomy, University of Otago, Dunedin, New Zealand; 2 Department of Anthropology and Archaeology, University of Otago, Dunedin, New Zealand; 3 Department of Biochemistry, University of Otago, Dunedin, New Zealand; 4 Department of Integrative Biology, University of California, Berkeley, United States of America; 5 Department of Anthropology, Southern Methodist University, Dallas, United States of America; 6 School of Geography, Archaeology and Environmental Studies, University of the Witwatersrand, Johannesburg, South Africa; Erasmus University Medical Center, NETHERLANDS

## Abstract

Dogs accompanied people in their migrations across the Pacific Ocean and ultimately reached New Zealand, which is the southern-most point of their oceanic distribution, around the beginning of the fourteenth century AD. Previous ancient DNA analyses of mitochondrial control region sequences indicated the New Zealand dog population included two lineages. We sequenced complete mitochondrial genomes of fourteen dogs from the colonisation era archaeological site of Wairau Bar and found five closely-related haplotypes. The limited number of mitochondrial lineages present at Wairau Bar suggests that the founding population may have comprised only a few dogs; or that the arriving dogs were closely related. For populations such as that at Wairau Bar, which stemmed from relatively recent migration events, control region sequences have insufficient power to address questions about population structure and founding events. Sequencing mitogenomes provided the opportunity to observe sufficient diversity to discriminate between individuals that would otherwise be assigned the same haplotype and to clarify their relationships with each other. Our results also support the proposition that at least one dispersal of dogs into the Pacific was via a south-western route through Indonesia.

## Introduction

Dogs are found in human communities throughout the world where they may be companions, working dogs or simply co-inhabitants of villages. They have a special place in human history as the first domesticated animal, having appeared in the archaeological record around 15,000 years ago, well in advance of other domesticates [1]. Since this time they have successfully moved with people across the globe, including through the islands of Oceania. Recent trends in dog keeping, particularly the rise in intensive breeding for particular physical characteristics, lie as a veneer over a much longer and more complex relationship between dogs and people.

**Fig 1:**
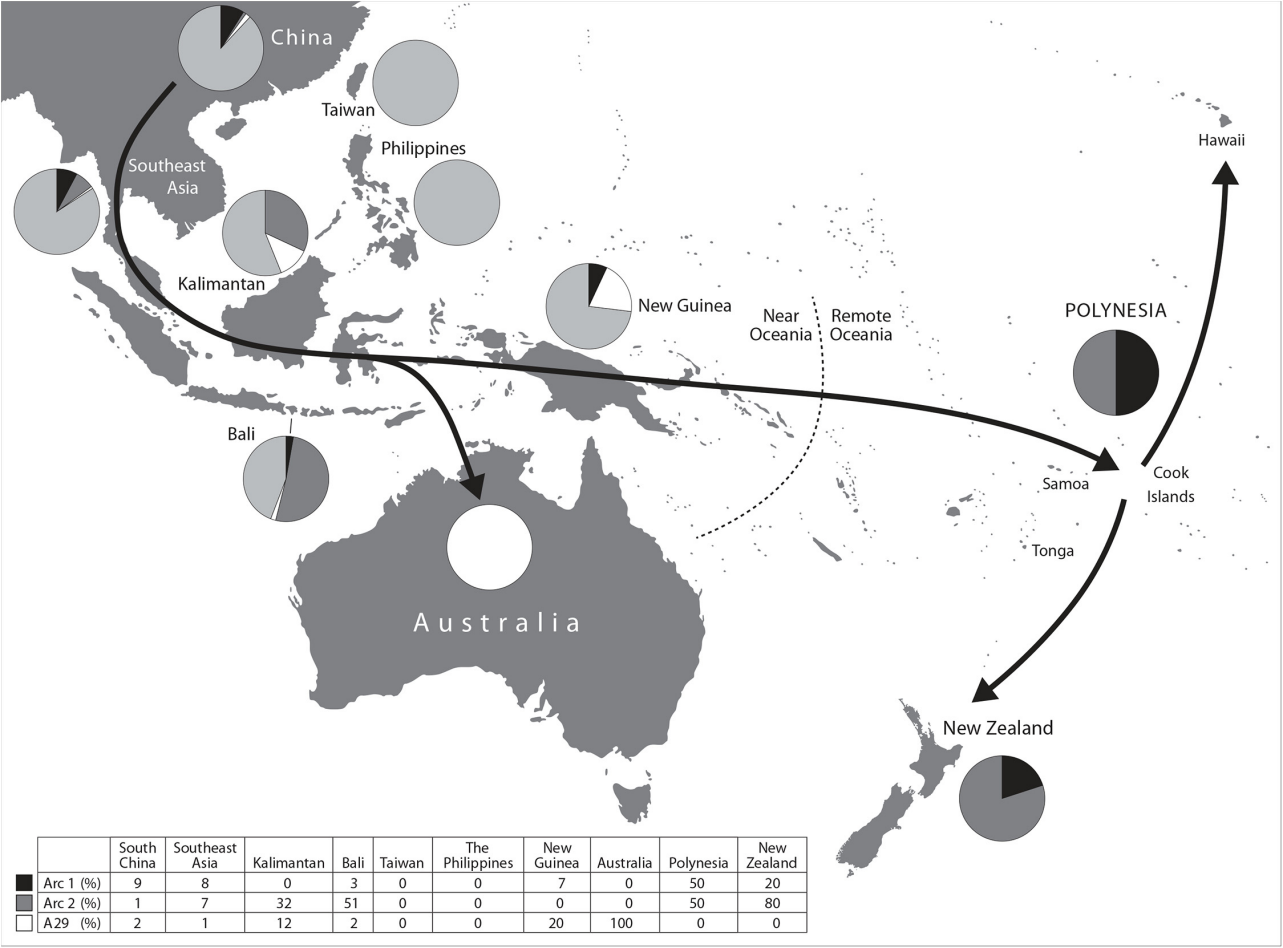
Map of the Pacific region. The boundary between Near and Remote Oceania is shown by the dotted line. Arrows indicate the proposed route for the introduction of dogs to Polynesia, via Island South East Asia. Pie charts and the summary table show the relative proportions of Arc1, Arc2 and A29 haplotypes observed in samples from across the region (after [4]).

Despite their long association with people, dogs appear relatively late in the archaeological record of Oceania (Fig 1). Near Oceania includes the large island of Papua New Guinea, the Bismarck Archipelago, and the Solomon Islands, and has evidence of human occupation that dates to as far back as the late Pleistocene (ca. 40,000 years ago). The antiquity of human occupation in Near Oceania contrasts with the relatively recent human colonisation of the islands of Remote Oceania to the north, east and southwest, which were not settled until about 3,000 years ago [2]. In addition to demarking two phases in the settlement of the region, the distinction also relates to differences in biogeography. The continental islands of Near Oceania possess the greatest biodiversity within the region. Further east across the Pacific the islands become smaller and more dispersed, and there is an accompanying drop in biodiversity [3].

The oldest evidence of dogs in Oceania comes from Australia, where the earliest dog (dingo) remains date from 3,500 years ago [5]. The dingo has subsequently adapted to surviving independently of people and is found in many areas of rural Australia. Although the dingo is considered a feral dog, individuals may also be taken in by indigenous communities where they become companions, protectors, and spiritual guardians, and can assist with hunting [6]. In Papua New Guinea, the New Guinea Singing dog (NGSD) is thought to be a descendant of another relatively early introduction, although no well dated or genetically confirmed archaeological NGSD remains have been reported. They are rarely observed in the wild and the current captive population is descended from only eight individuals [7]. In Australia, New Guinea, and the islands of Near Oceania, dogs were introduced to areas where people had already been living for thousands of years.

The introduction of dogs into Remote Oceania is associated with the migration of people to these previously uninhabited islands, which began about 3,350 years ago [2]. This process is marked by the appearance of a distinctive pottery, termed ‘Lapita’, which is present in numerous archaeological sites that extend from Papua New Guinea to the western margins of Polynesia. The presence of Lapita pottery has been linked to the arrival of people originating from Island South East Asia, who spoke Austronesian languages. In addition to pottery and languages, these people introduced agricultural practices, a suite of domesticated animals and plants, a settlement pattern of villages situated on intertidal reefs or small offshore islands, and a characteristic set of artefacts including adzes and shell ornaments [8]. Dogs are generally considered to be one of the Lapita domesticated animals, along with pigs and chickens. To date though, Lapita dog remains are relatively rare, having only been recorded in limited numbers in archaeological sites in the Bismarck Archipelago of New Guinea (Table 10.1 in [9]) and have not been reported in any of the key archaeological sites in Vanuatu or New Caledonia.

The subsequent, post-Lapita settlement of the islands further to the east also involved dogs, pigs and chickens, although they were not uniformly distributed across the region. The settlement of Polynesia occurred in two phases, beginning with the islands of West Polynesia around 3,000 years ago during the Lapita expansion. Then after a substantial hiatus, East Polynesia was rapidly settled over several hundred years following the arrival of people initially to the central tropical islands about 1000 years ago [10]. This second settlement phase included the islands at the margins of East Polynesia: Hawaii, Rapa Nui and New Zealand. In some of these island groups, such as Hawaii and New Zealand, the large number of dog bones found in early archaeological sites contrast strongly with the limited evidence of dog remains in the Lapita archaeological sites further west.

By the time European explorers arrived in Polynesia in the eighteenth century, dogs were present on some but not all island groups. In East Polynesia, dogs were often an important part of the social and economic fabric of daily life [11]. In the ranked societies of Hawaii, dogs were observed as the property of chiefs and were raised in large numbers for feasts. In New Zealand, dogs were the only domesticated animal to be successfully introduced by the Polynesian ancestors of the Maori. While living, they were kept as watch-dogs, hunting dogs and general companions and were sometimes also kept for their hair; on their death they could be used as food for ceremonial occasions, their bones and teeth as industrial materials, and their pelts to make dog skin cloaks [12].

Previous studies using mtDNA to investigate the origins and dispersals of the dog throughout the Pacific indicate that the dingo, NGSD and Polynesian dogs are all descended from East Asian dogs (Fig 1). Using a 582 base pair (bp) fragment of the control region of the mitogenome (mitochondrial genome) from modern dogs, Savolainen and colleagues [13] demonstrated that all dingoes sampled belonged to the A29 haplotype. Although this is one of a number of dog mtDNA lineages that reached Island Southeast Asia (ISEA) it was the only one successfully established in Australia. On this basis, they suggest that the dingo population was either founded by a very small number of individuals, or from a group of dogs that had passed through a series of genetic bottlenecks and hence had lost substantial genetic variation. Savolainen and colleagues [13] also reported that the 19 ancient Polynesian dogs samples, based on analysis of a shorter 263 bp control region fragment, belonged to two short haplotypes, Arc1 and Arc2, both different from the A29 control region haplotype carried by dingoes. The short Arc1 haplotype is indistinguishable from a number of widespread control region haplotypes, while Arc2 appears to belong to the A75 lineage found in modern Indonesian dogs.

A later study by Oskarsson and colleagues [4] investigated the origins and routes of the introductions of dingoes, NGSDs, and Polynesian dogs in further detail, using the same 582 bp and 263 bp Arc1 and Arc2 control region fragments. The study used samples collected from modern dogs and dingoes, previously published modern dog and dingo sequences, and the ancient Polynesian short sequences. Oskarsson and colleagues [4] compared the frequency and distribution of modern haplotypes in Mainland and Island Southeast Asia, including Taiwan and the Philippines, with those from the dingo and ancient Polynesian samples (Fig 1). Arc1 and Arc2 short haplotypes were found in 10% of modern dogs samples in South China, 16% in Mainland South East Asian dogs, and 42% in Indonesian dogs. The dingo haplotype A29, was found in 2% of modern dogs sampled in South China, 1% of dogs in Mainland South East Asia, and 8% of Indonesian dogs. The short Arc1 haplotype appears to have a predominantly mainland and western Island South East Asia distribution, while Arc2 is found in increasing frequency across Island South East Asia. Additionally, they found that the short Arc1 haplotype corresponded to a possible 13 control region haplotypes, while Arc2 was limited to a possible two, A75 and A120. Neither the ancient Polynesian short haplotypes nor the A29 haplotype carried by dingoes, were present in dogs from Taiwan and the Philippines, suggesting that dogs were not introduced into the Pacific region from or via this northeastern route.

Following recent advances in sequencing technology we can now use next generation sequencing to obtain complete ancient mitogenomes. As would be expected, recently published studies of complete mitogenomes of cattle [14] and sheep [15] have shown that the control region sequences provide only a partial picture of genetic diversity. This has also been the case in a recent study of mitogenomes from four human burials at Wairau Bar, which demonstrated that the people buried there possessed greater than expected mtDNA diversity [16]. The ability to observe a more fine-grained level of diversity can be particularly significant when addressing founding population structure, particularly in a geographic area with a settlement history such as that in the Pacific, which most likely involved population bottlenecks and a relatively short chronology of human occupation.

Here we describe complete mitochondrial genome sequences for fourteen dogs from the site of Wairau Bar, one of the earliest archaeological sites in New Zealand and probably a central place during the colonisation phase [17]. Wairau Bar comprises a settlement with middens, living floors, specialist work zones, food preparation areas, and a burial ground. The site also contains numerous bones of moa, other extinct birds, and a suite of artefact types that link New Zealand with the tropical East Polynesian homeland zone. Because Wairau Bar is among the earliest settlement sites in New Zealand, it is a critical place for understanding the colonisation processes of the archipelago including the successful introduction of dogs to the southern-most point of their Oceanic distribution.

## Materials and Methods

### Samples

**Fig 2:**
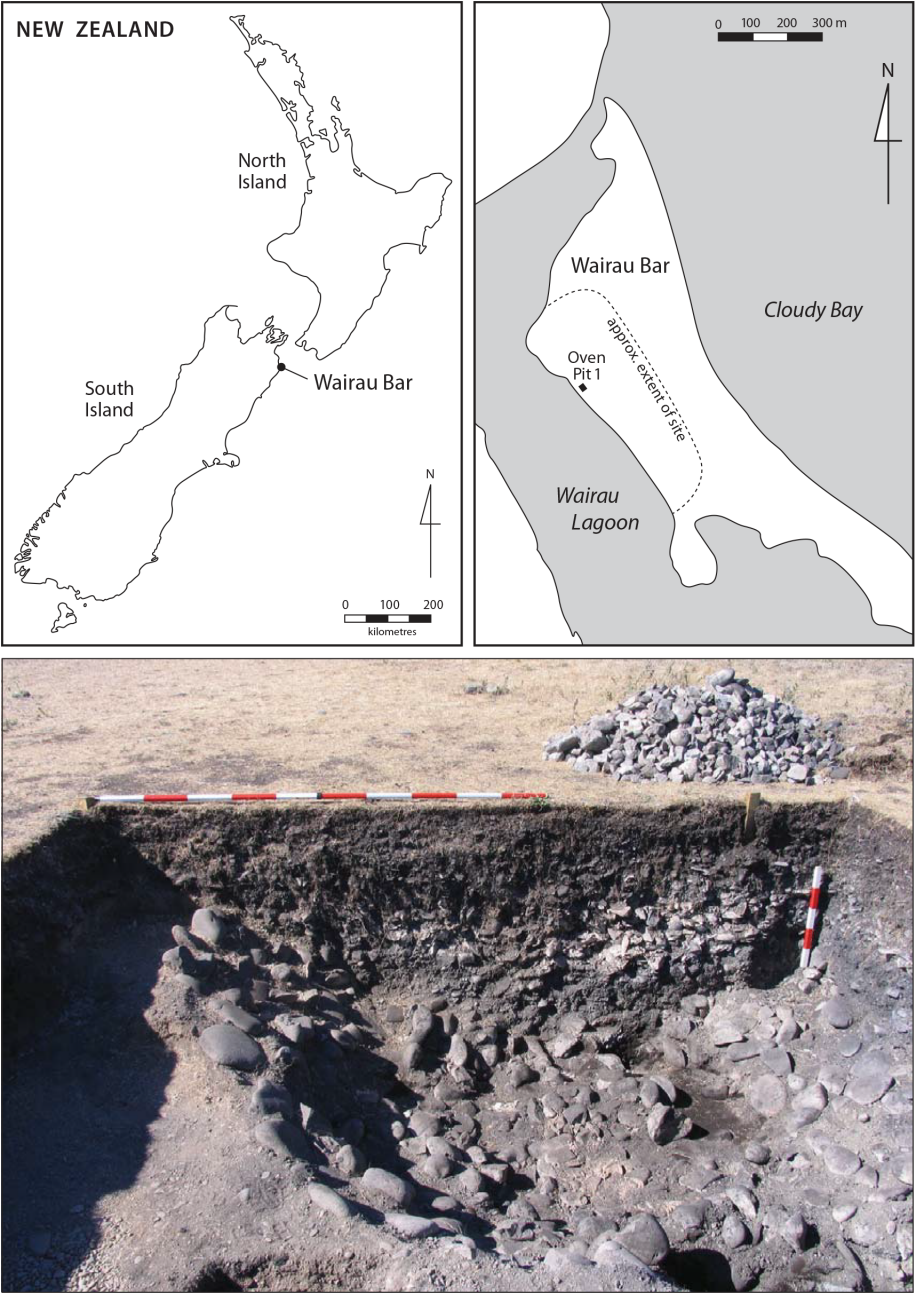
Wairau Bar archaeological site. Location of the archaeological site (top left), location of Oven Pit 1 (top right), and section view of Oven Pit 1 (horizontal scale 2 m, vertical scale 0.5 m).

Dog teeth were selected from faunal material recovered from a large cooking feature (Oven Pit 1) during archaeological excavations in 2009 [17] (Fig 2, S1 Table). These excavations were fully authorised by the appropriate government agencies (NZ Historic Places Trust Authority No. 2009/121), and the excavated faunal collections are held at the Otago Archaeological Laboratories, University of Otago, Dunedin, New Zealand. The oven fill contained bone and shell midden, charcoal, and fire-cracked rocks. In addition to dog, fauna identified from the oven included the extinct moa (bone and eggshell) and at least 60 species of other birds, marine mammals, fish and shellfish. Moa eggshell from Oven Pit 1 was used to develop a detailed chronology drawing on ancient DNA, and Bayesian analysis of radiocarbon dates, which showed the oven had been filled with midden during a single event that occurred sometime after the early 1320s and no later than about 1350 [17]. The rapid deposition of the oven fill indicates that the dogs were all likely to have been a part of the same dog population living at Wairau Bar immediately prior to their death and incorporation in the midden. Over 400 dog bones, teeth and bone fragments were excavated from the oven, which represents at least 21 dogs. The large number of dog bones excavated from the site allowed 16 specimens of the same element (left maxillary fourth premolar) for inclusion in this study. This means that each of the 16 specimens represents a different individual, and ensures that no dog was sampled more than once.

### Extraction, hybridisation capture and sequencing

All DNA extraction and sequencing library preparation before PCR amplification was carried out at the Ancient DNA Laboratory at the University of Otago, where stringent procedures are in place to avoid contamination [18]. DNA extraction was performed on sixteen teeth. To reduce surface contamination, the teeth were submerged in 6% bleach for ten minutes and rinsed in ultra pure water prior to grinding for DNA extraction. Teeth were ground using a mortar and pestle and 150 to 250 mg was used for extraction following a silica based extraction protocol [19]. At least one extraction blank was processed in parallel with each set of five samples. Extraction blanks were then treated as samples throughout processing and sequencing.

Barcoded sequencing libraries were prepared directly from the DNA extract as described by Knapp and colleagues [20] for Illumina sequencing, with the following modifications. Quantitative PCR (Stratagene MxPro 3000P) was used to determine the number of cycles necessary to reach amplification plateau. Libraries were immortalised by PCR amplification to plateau using ABI’s AmpliTaq Gold with the following reagent concentrations: 1x AmpliTaq PCR Buffer, 2.5 mM MgCl2, 1 mM dNTPs, 0.2 μM of each extension primer and 3.75 units of AmpliTaq Gold. Amplifications were stopped when PCR plateau was reached to avoid the manufacture of chimeric molecules [21–24]. Immortalised libraries were purified using MinElute columns (Qiagen) following the manufacturer’s protocol with the addition of a second PE wash and a 5 minute incubation with 0.1x TE buffer instead of the provided elution buffer.

Dog mtDNA was enriched in the libraries by in-solution hybridisation capture [25], with the following modifications. One PCR of 1 μl barcoded library in a 50μl reaction volume were performed for each sample prior to capture to obtain 2μg of sequencing library per sample, using KAPA’s HiFi DNA Polymerase with the following reagent concentrations: 1x KAPA HiFi Buffer, 0.3mM of each dNTP, 0.3μM of each primer, and one unit of KAPA HiFi DNA Polymerase. Each sequencing library was enriched independently, and pooled in equimolar ratios after a further 10 cycles of PCR amplification. Additionally, the libraries were eluted from the MyOne Streptavidin C1 Dynabeads (Invitrogen) by heating for 3 minutes to 95°C instead of treatment with sodium hydroxide. Libraries were quantified using the Qubit 3.0 fluorometer (Life Technologies), pooled in equimolar concentrations and sequenced using 2 x 75 base pair-end runs on the Illumina MiSeq sequencing platform. Libraries prepared from extraction blanks were subsequently sequenced on a separate run. In addition, the bait used for capture (tissue obtained from a local veterinary clinic) was separately sequenced and checked against sample sequences at variable positions.

### Raw Data Processing and Analysis

As ancient DNA fragments are often short enough to contain the sequencing adaptor, we pre-processed the raw reads to remove adaptors and merge paired end fragments, which overlapped by at least 11 base-pairs, using AdapterRemoval (v1.5.4) [26]. Reads were also processed to remove stretches of Ns, bases that had a low quality score (<30), and short reads (<25).

Contamination from modern DNA represents a challenge for any ancient DNA study, particularly when archaeological samples were not collected specifically for aDNA analyses, as sequenced reads from modern organisms may map to the reference genome of interest. To identify the impact of this effect, all read mapping was done onto a composite reference genome consisting of the Cambridge Reference Sequence for humans [27], in addition to mitochondrial reference genomes from cow (*Bos taurus*, Gen Bank NC_006853), pig (*Sus scrofa*, NC_0012095.1), and chicken (*Gallus gallus*, Gen Bank NC_001323.1). The contamination ratio was determined by calculating the ratio of all reads with mapping quality > = 20 for each reference, compared with those for the dog mitochondrial reference sequence (NC_002008) [28].

Ancient DNA is usually characterised by C→T transitions at the 5' ends of the molecule, and following double stranded library preparation G→A transitions at the 3' ends of the molecules [29]. Reads were aligned to the composite reference genome using BWA (0.7.10) [30] with recommended ancient DNA settings [31]; specifically, seeding was disabled (-l 1024), the number of gap opens was set to 2 (-o 2), and the maximum edit distance was set to 0.03 (-n 0.03). PCR duplicates were removed from the merged reads using a python script originally developed for the Neanderthal genome project [32]. These PCR duplicates were also removed from the unmerged reads using Picard’s MarkDuplicates tool (http://broadinstitute.github.io/picard/). To confirm the authenticity of our ancient DNA, the program mapDamage (v2.0.2–9) [33], which identifies characteristic aDNA patterns, was used with the '-rescale' option to lower the quality score of likely damaged sites. The plots of characteristic damage patterns were individually created for merged and unmerged reads. Finally, Ts (thymines) found at the 5' end of a read and Gs (guanines) at the 3' end of the read, within the first two bases, had their quality scores rescaled to zero. Samples with less than 95% coverage of the dog reference genome were removed, as retaining samples where the DNA only partially covers the dog reference genome would have significantly impacted downstream analyses [34].

Reads that mapped to the dog reference genome were extracted from the composite alignment and variant call files (VCFs) were generated using the GATK Haplotype Caller (v3.3) [35] with settings specific for haploid genomes, such as the mitochondrial genome. This file was filtered for mapping quality (<20), and bases supported by fewer than three reads [16, 36]. This approach was justified under the assumption that the above quality control (QC) procedure preferentially reduces the quality of the damaged positions in the sequencing reads, which would lead to a reduction in the probability that the damaged position would be called downstream. Finally, to maximise the usability of all the sequences obtained in our analyses, we carried out imputation using Beagle (v4.0 r1399) with default settings and the 'gt' argument for the VCF input, imputed sites with a probability greater than 0.8 were retained [37]. To confirm the results of the QC, and imputation, visual inspection of all sites in the alignment files was undertaken using IGV [38]. The imputed VCFs were then compared with the positions in the bait sequence that varied from the reference. The bait sequence was processed separately using the same computational protocol as the Wairau Bar samples. In addition, libraries prepared from negative controls were processed and aligned to the composite reference genome.

Coverage plots were created for all samples, and plots of fragment length were created for the merged reads using the ggplot2 package for the R programming language [39, 40].

Consensus sequences containing indels (insertions and/or deletions) were created for each sample that passed QC, and these have been deposited in GenBank (Accession Numbers KT168369-KT168382). In addition to the aforementioned QC measures, for these GenBank sequences non-variant sites that were supported by fewer than three reads were changed to 'Ns'.

### Phylogenetic Analyses

The population structure of the Wairau Bar dogs was characterised using network analysis. Networks usefully visualise the relationships between haplotypes, and can help to understand how haplotypes are related to each other [41]. The imputed VCF files were converted to FASTA and then to NEXUS format, using a custom python script and Biopython [42] respectively. This dataset was then used to create a median-joining network using Popart (v1.7.1) (http://popart.otago.ac.nz/) with default settings. This method works by resampling from clusters of trees that minimise the distance between haplotypes to generate a parsimonious network.

To investigate the place of the ancient New Zealand dogs in wider regional and global dog populations, we combined the ancient DNA sequences from the Wairau Bar dogs with sequences reported from previous studies of ancient and modern dogs. To date, the only reported ancient dog sequences from the Pacific comprise very short fragments of the control region from Polynesian and New Zealand dogs [13]. Thalmann and colleagues [36] have recently documented complete mitogenomes for ancient dogs. Although these samples came from archaeological contexts in Europe and North America we have included samples from the major dog haplogroup A in our analyses as they provide the only other published ancient mitogenomes for comparison with the Wairau Bar dogs. Mitochondrial genome sequences and control region haplotypes were also obtained from studies of modern dogs from the Asia-Pacific region [4, 13, 43] to investigate the geographical context of the samples.

We used the multiple sequence aligner MUSCLE (v3.8.31) [44] to align the Wairau Bar sequences to the ancient and modern mitogenomes, and the modern control region haplotypes obtained from the literature. In their study of mitogenomes from dogs and wolves worldwide, Pang and colleagues [43] analysed only 16,195 bp of the mitochondrial genome to allow them to exclude repetitive and difficult-to-align regions. The control region (15458–16039) was extracted from Wairau Bar sequences using bioawk (https://github.com/lh3/bioawk) for comparison with the modern control region haplotypes.

Median joining networks were then created with these datasets. In addition, the ancient Polynesian short control region fragment (15458–15720) was extracted from the Wairau Bar sequences and compared to the Arc1 and Arc2 haplotypes to investigate if they are present within our dataset ([13], Supplementary material).

## Results

### DNA preservation and sequence recovery

Acceptable sequence data was obtained from fourteen of the sixteen samples from which libraries were prepared (S1 Table). Two samples failed to generate acceptable consensus sequences (greater than 95% of the reference sequence covered by a read depth of greater than two) so were discarded from further analyses. Complete mitogenomes were obtained from three specimens, with the remaining eleven specimens having nearly complete coverage (S1 Fig). The control region had proportionally lower read depth than elsewhere in the mitogenome in all the samples (S2 Fig), which may be related in part to the presence of a repetitive region confounding capture and sequencing in these regions [43]. The median average read depth for the fourteen samples was 86x (varying across samples from between 6x to 424x (S1 Table). The two discarded samples, MS10064 and MS10067, had much reduced coverage in comparison with other samples and also substantially lower read depth.

These results are significantly better than those reported for the human remains from Wairau Bar, where only four out of nineteen individuals provided sufficient mtDNA for downstream analyses [16]. This may be related to two factors; firstly, improved methods (Illumina vs. Roche 454 sequencing platforms], and secondly, that the dog remains were excavated very recently. The human remains were excavated over forty years ago, and had been housed in a museum collection prior to ancient DNA extraction.

### Sequence authenticity

Throughout DNA extraction and library preparation, blank extractions were processed alongside samples to provide negative controls. None of the controls contained any reads mapping to the dog reference mitogenome. The possibility of contamination due to laboratory reagents has also been raised in ancient DNA studies, based on the results presented by Leonard and colleagues [45]. Our analyses show very low levels of human and cow contamination in the dog samples (S2 Table). No chicken DNA was found in any of the samples. Additionally, our analyses showed that only six of the 479,410 reads from the blank extraction libraries mapped confidently (MAPQ > 20) to the composite reference genome containing mitochondrial DNA from human, dog, pig, and chicken. No reads supporting variable positions in the bait sequence were detected. Damage patterns such as short fragment lengths (S3 Fig) and deamination patterns (S1 File) are consistent with those expected from aDNA [46].

### Population structure of Wairau Bar dogs

**Fig 3:**
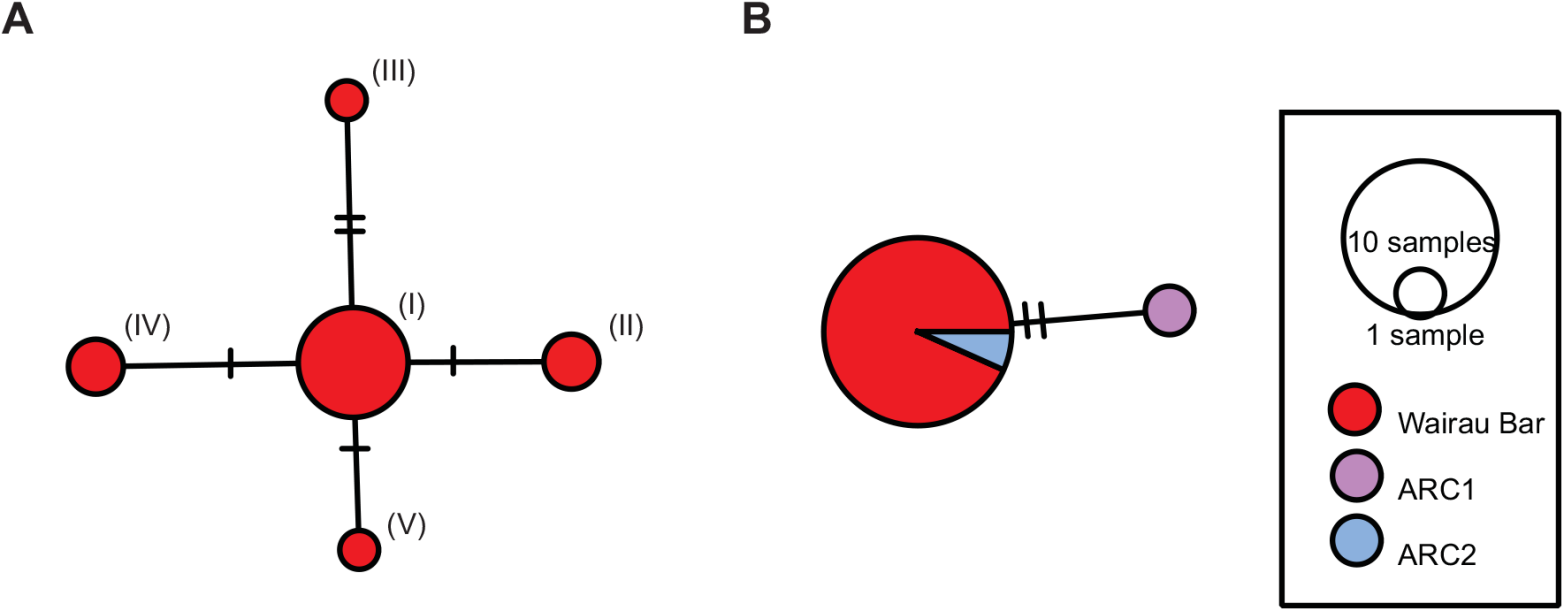
Median-joining networks for Wairau Bar dog sequences. a) Complete mitochondrial genomes. Sample numbers: (I) MS10062, MS10068, MS10070, MS10130, MS10131, MS10132, MS10135, MS10137, (II) MS10065, MS10136, (III) MS10069, (IV) MS10129, MS10133, (V) MS10066. b) Comparison with 263 bp Arc1 and Arc2 short control region fragment.

The median-joining network constructed for the Wairau Bar dogs (Fig 3) has a single main node that comprises eight samples, with four nodes radiating outwards. Two of those nodes contain two samples each, separated by one mutation. Another single sample differs from the central node by only one mutation. There is also a further single sample that is separated from the central node by two mutations. The network illustrates the low level of genetic diversity among the dogs we have sampled from Wairau Bar, with five haplotypes spread across the fourteen specimens. These five haplotypes are closely related to one another, differing by a maximum of three mutations across 16.7kb of the mitogenome and the entire network. The central node contains over half of all the dogs sampled.

Since the archaeological site at Wairau Bar is one of the earliest sites in New Zealand, sufficient time is highly unlikely to have elapsed since settlement for new lineages to arise within the dog population. It is possible therefore that the haplotypes observed in the dogs we have sampled represent the founding lineages for the initial Polynesian dog population in New Zealand. Determining the number of founding haplotypes based on ancient DNA can, however, be a challenging task as observed variation can be the result of private mutations or false positives resulting from DNA damaged variants being called [47]. In addition, given the tight temporal and spatial context of the sample (all individuals came from the same oven feature, deposited in one event) samples may represent several generations carrying the same lineage, possibly bitches and their offspring. This tight context may constrain our ability to identify founding lineages from within the wider Wairau Bar dog population.

### Complete mitogenomes

**Fig 4:**
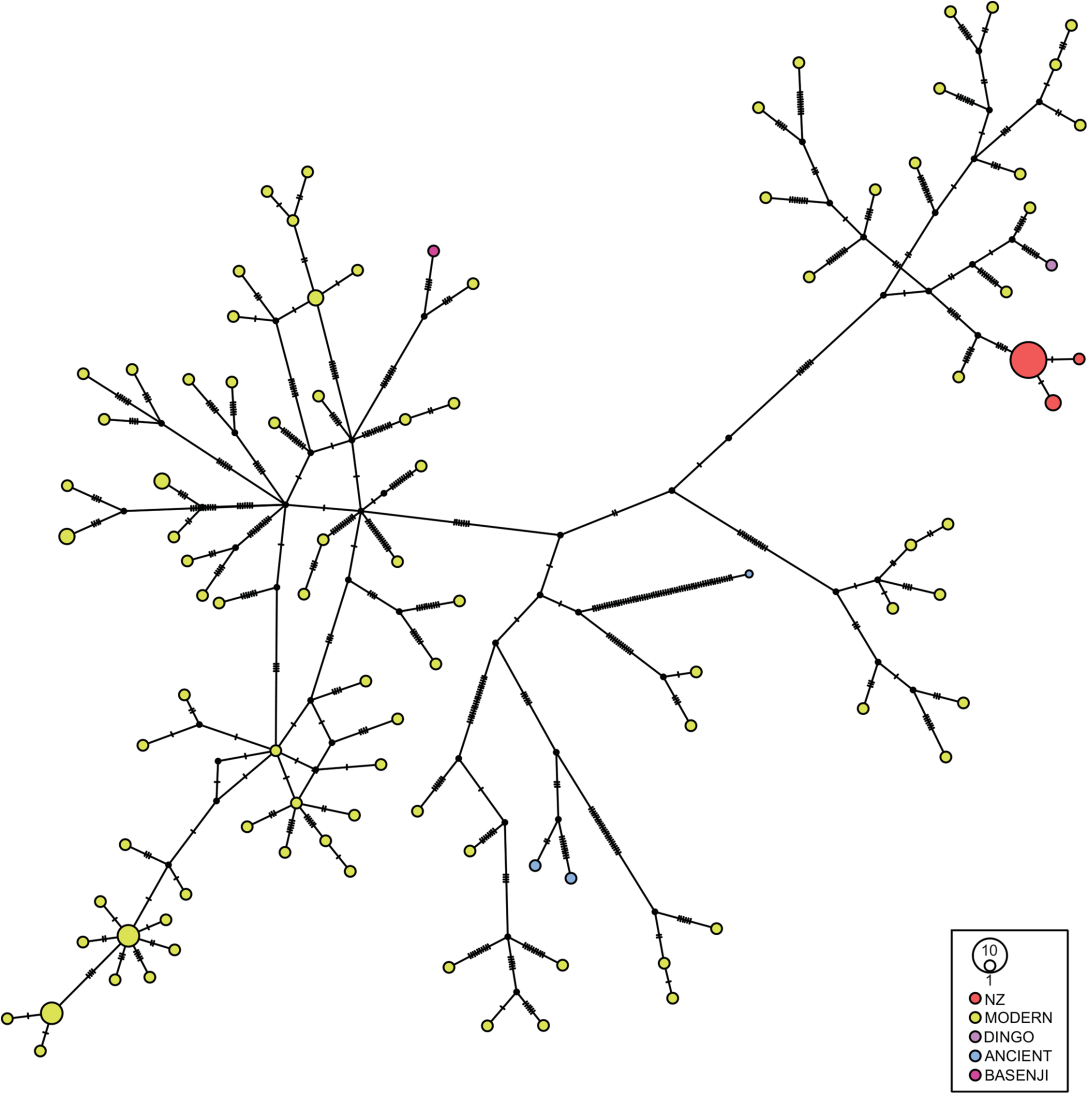
Median-joining network of complete mitochondrial genomes of Wairau Bar dogs, and modern and ancient sequences from Haplogroup A. Wairau Bar = red, Dingo = purple, ancient American dogs = blue, Basenji = pink.

The network in Fig 4 shows the Wairau Bar dogs along with published ancient mitogenomes from the Americas, ancient breeds the Dingo and Basenji [36] and modern East Asian dogs [43]. The ancient Wairau Bar dogs form a cluster that is located on a major branch of the network, while the Basenji and other ancient dogs are on other branches. The major Wairau Bar dog branch, which has two other branches one of which includes the Dingo sample, corresponds to Pang and colleagues’ [43] sub-group a2 for Haplogroup A, which has a predominantly East Asian distribution. The modern individual on the same branch as the Wairau Bar dogs comes from a dog sampled in Thailand that carries the A75 control region haplotype.

### Control region haplotypes

In the absence of complete mitogenome sequences from ancient dogs from the Asia-Pacific region, we compared sequences from the Wairau Bar dogs with published control region haplotypes (582 bp, positions 15,458–16,039) for modern dogs within the dog haplogroup A [4, 43]. These control region haplotypes have been used extensively for studies of dog domestication and dispersal throughout the Old and New Worlds. The Wairau Bar dogs all carry the control region haplotype A192. This haplotype has only been reported in modern village dogs from Bali in Indonesia [4].

**Fig 5:**
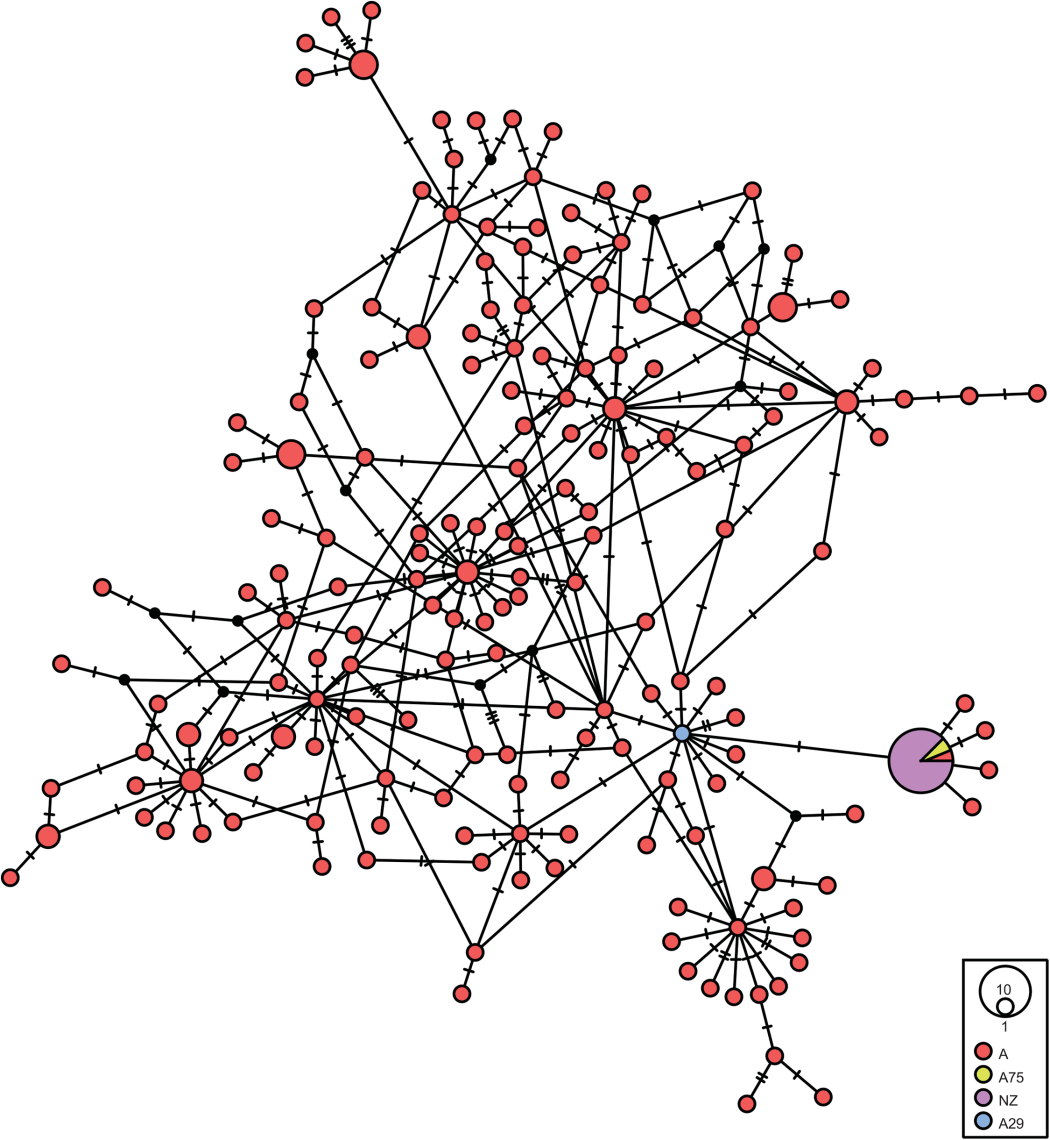
Median-joining network of control region sequences (582bp) of Wairau Bar dogs, and modern sequences from Haplogroup A. Modern = red, Wairau Bar = purple, A75 = yellow, A29 = blue.

The network in Fig 5 was constructed from control region sequences from the Wairau Bar dogs and modern Haplogroup A dog sequences, ignoring insertion or deletion mutations (indels) which are not taken into account in network construction. The Wairau Bar dogs form a node with the A75 and A192 haplotypes. The A192 haplotype contains several indels. If the indels are discounted, the A192 haplotype corresponds to A75 ([4], Supplementary material). This A75 haplotype has been found in modern dogs in small numbers in China and Thailand, and in much higher frequencies in Indonesia (40%) [4]. Also expanding off this node are two haplotypes (A194, A195) also obtained from samples of modern dogs from Bali, and one (A145) reported from China and Bali [4].

When compared with the Arc1 and Arc2 short fragment reported for 19 ancient Polynesian dog samples, generated by the Matisoo-Smith laboratory [13], all the Wairau Bar dogs produced sequences that would be assigned to the short haplotype Arc2 (Fig 2B, S3 Table). This haplotype occurs in modern dogs throughout Mainland and Island South East Asia [4, 13]. To date, the Arc1 haplotype has not been identified in any dogs from Wairau Bar, either as part of the short fragment study [13], or during this study. The Arc1 haplotype observed in the short fragment study was present in dogs from three New Zealand archaeological sites, at Otehei Bay, Urupukapuka Island, Bay of Islands, Harataonga, Great Barrier Island, and Redcliffs, Christchurch, mainland South Island (Matisoo-Smith, unpublished data).

## Discussion

Dogs from Wairau Bar likely represent part of the initial population of dogs introduced to New Zealand, having arrived with people around the beginning of the fourteenth century AD [17]. We sequenced fourteen complete or nearly complete mitogenomes, from a sample of dog teeth obtained from one oven feature at this important site. We observed five different mitochondrial haplotypes, which may have contributed to the establishment of the dog population of New Zealand. While it is possible that these haplotypes represent private mutations that are not carried further in the dog population, they may also be founding lineages. Further analyses of dogs from early archaeological sites in New Zealand would assist with determining the status of these lineages, if any additional haplotypes were introduced elsewhere, or if the Wairau Bar dog population can be considered representative of the Polynesian derived founding population as a whole. This initial dog population was then subsumed by a new wave of dogs introduced during the European colonisation of New Zealand in the 19^th^ century. Analyses of dog bone from archaeological sites provide the only method of investigating the arrival and dispersal of the first dogs to arrive in New Zealand.

The limited number of mitochondrial lineages present at Wairau Bar suggests that only a few dogs may have been introduced to New Zealand within the first few years of settlement, despite the large numbers of dog bones recovered from early archaeological sites throughout the country [12]. However, it is also possible that the founding population came from a source population with very limited diversity, or that a combination of both factors contributed to the observed lack of variation. Historical observations of animal management practices in New Zealand and elsewhere in Polynesia, and the lack of animal control measures visible in the archaeological record, indicate that the majority of the dogs at Wairau Bar and other early archaeological sites were likely to be free ranging. The carrying capacity of free ranging dogs is closely related to the human environment that they occupy [48], and it is possible that a large dog population grew rapidly from a small number of dogs following their arrival in New Zealand, given the quantity and quality of food sources available.

For populations such as that of Wairau Bar, which represent relatively recent migration events, the use of data solely from the mitochondrial control region is insufficient to address questions about population structure and founding events. Our analyses demonstrate the benefits of adopting a more fine-grained approach that identifies sufficient diversity to discriminate between individuals that would otherwise be assigned the same haplotype, and to clarify their relationships with each other. As with the dingo in Australia [13], the founding population of dogs in New Zealand is likely to have been drawn from a group of dogs that had passed through a series of genetic bottlenecks at times during their movement across the Pacific, hence arriving in New Zealand with limited genetic variation. The use of mitogenomes, however, enables us to identify five haplotypes within the Wairau Bar sample, whereas only one (A75) could have been identified had we used only the control region.

The analysis of mitogenomes of the Wairau Bar dogs also contributes to our understanding of the dispersal of dogs throughout the Asia-Pacific region, prior to their arrival in New Zealand. Pang and colleagues’ [43] analyses of the major dog clade identified ten sub-groups within Haplogroup A, with distinct geographical patterning. However, the majority of published mitogenomes from their study were from mainland China, with a few from Thailand, but none from Island South East Asia. The Wairau Bar dogs' mitogenomes sit within sub-group a2, which has an East Asian distribution and includes the A29 control region haplotype found in dogs and dingoes [43]. Further analyses of full mitogenomes from Island South East Asian village dogs and ancient DNA analyses of dog bone from archaeological sites could assist with understanding the distribution of dog lineages in this region, as human migrations from Island South East Asia likely played a pivotal role in the dispersal of dogs out into the Pacific.

The Wairau Bar dogs all carry the control region haplotype A75 (if indels are included in the analysis, the Wairau Bar dogs correspond to the control region haplotype A192) and the short Arc2 haplotype. The A75 control region haplotype and the Arc2 short haplotype have a distribution across Mainland China, Island South East Asia and the Pacific, but to date have not been found in modern dogs in Taiwan or the Philippines [4]. The A192 haplotype is found in modern village dogs from Bali, Indonesia ([4] Supplementary Information). The settlement of Remote Oceania is associated with expansion of Austronesian language speakers; these languages are thought to have originated in Taiwan, and were then introduced through the Philippines and southwards through Island Southeast Asia and into the Pacific [49]. Based on the presence of the A75 (A192) haplotype in the Wairau Bar dogs and the modern distribution of this haplotype, it appears that at least one of dog dispersals into the Pacific followed a more south-western route through Indonesia. Interestingly, this has parallels with the early movement of pigs and rats, based on genetic and geometric morphometric studies [50, 51].

The short haplotype Arc2 was carried by all of the Wairau Bar dogs analysed in our study. Previous short fragment analysis of other ancient samples from New Zealand and Polynesia found that samples were split between two haplotypes, Arc1 (32%) and Arc2 (68%). At this point it is not known whether the lack of the Arc1 haplotype arises from a sampling bias, or relates to geographical or chronological factors. The Arc1 haplotype has been previously identified in dog samples from three archaeological sites in New Zealand, two of which are offshore islands (Urupukapuka and Harataonga). Further analyses are required to investigate the possible distribution of Arc1 in New Zealand.

We sequenced fourteen complete mitogenomes of dogs from one of the earliest archaeological sites in New Zealand and were able to identify five haplotypes, rather than the single haplotype that would have been observable using the control region. The arrival of people and dogs in New Zealand represents the end point of the last major pre-industrial human migration; yet questions still remain about the timing and routes taken. While the preservation conditions in the Pacific are generally poor, particularly for open coastal sites like Wairau Bar, our results indicate that obtaining nuclear data from the best preserved of these samples may be possible in the future. Such data would allow us to address a range of questions including population structure among closely related individuals, and human selection for particular traits. The sequencing of additional complete mitogenomes from dog bones from New Zealand and Polynesian archaeological sites offers the possibility of refining our understanding of the dispersal of dogs during this last major human migration, and their subsequent movements at local and regional scales. Our results have wider implications for the use of dogs in studies about human migrations in other regions, since the sequencing of mitogenomes may greatly enhance the information potential of archaeological dog remains.

## Supporting Information

S1 Fig Graph of mitogenome coverage of the Wairau Bar dog sequences. Each sample is represented by a horizontal bar. The length of each bar on the x-axis shows the proportion of the mitochondrial genome covered by at least one read. The read depth is shown by the colour gradient given in the legend.
(PNG)

S2 Fig Read depth for Wairau Bar dog sequences. The x-axis represents the length of the dog mitochondrial genome in base pairs. The y-axis is the number of reads covering each site of the mitogenome.
(PNG)

S3 Fig Size distribution of merged endogenous mtDNA fragments from Wairau Bar dogs. Number of reads and fragment length are provided.
(PNG)

S1 File Wairau Bar dog sample damage patterns. Top plots: Base frequency 5′ and 3′ of strand breaks. The gray brackets indicate start and end of molecules (strand breaks). Purines (A and G) show an elevated frequency before strand breaks. Bottom plots: C to T and G to A nucleotide misincorporations at the first and last 25 bases of endogeneous mtDNA fragments from the Wairau Bar dog sample [46].
(PDF)

S1 Table Wairau Bar dog sample showing specimens used for analyses, GenBank Accession Numbers, coverage and read depth statistical summary, and percentage of imputed bases. (DOCX)

S2 Table Levels of contamination observed in Wairau Bar dog samples. (DOCX)

S3 Table Informative sites for Arc1 and Arc2 short control region fragment. (DOCX)
