# Supplementary figures and images for: Complete Mitochondrial Genomes of New Zealand’s First Dogs
Source: PLoS One. 2015 Oct 7;10(10):e0138536. doi: 10.1371/journal.pone.0138536 (PMC4596854; doi:10.1371/journal.pone.0138536)

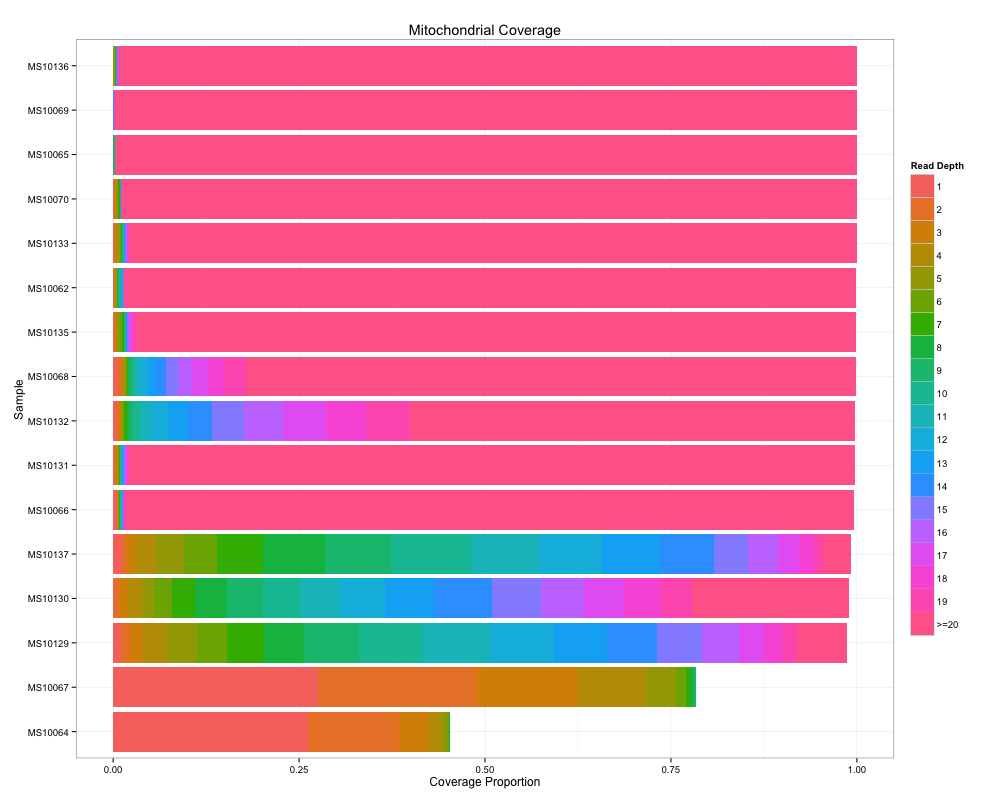

Supplement: S1 Fig — Each sample is represented by a horizontal bar. The length of each bar on the x-axis shows the proportion of the mitochondrial genome covered by at least one read. The read depth is shown by the colour gradient given in the legend. (PNG) [file pone.0138536.s001.png]

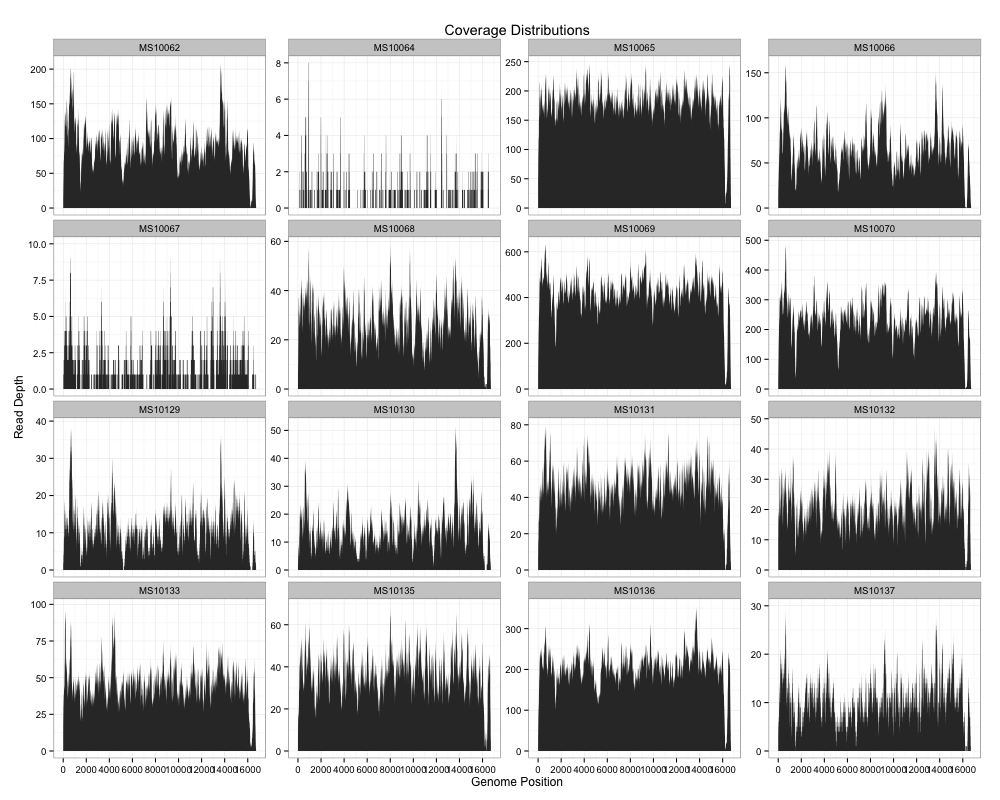

Supplement: S2 Fig — The x-axis represents the length of the dog mitochondrial genome in base pairs. The y-axis is the number of reads covering each site of the mitogenome. (PNG) [file pone.0138536.s002.png]

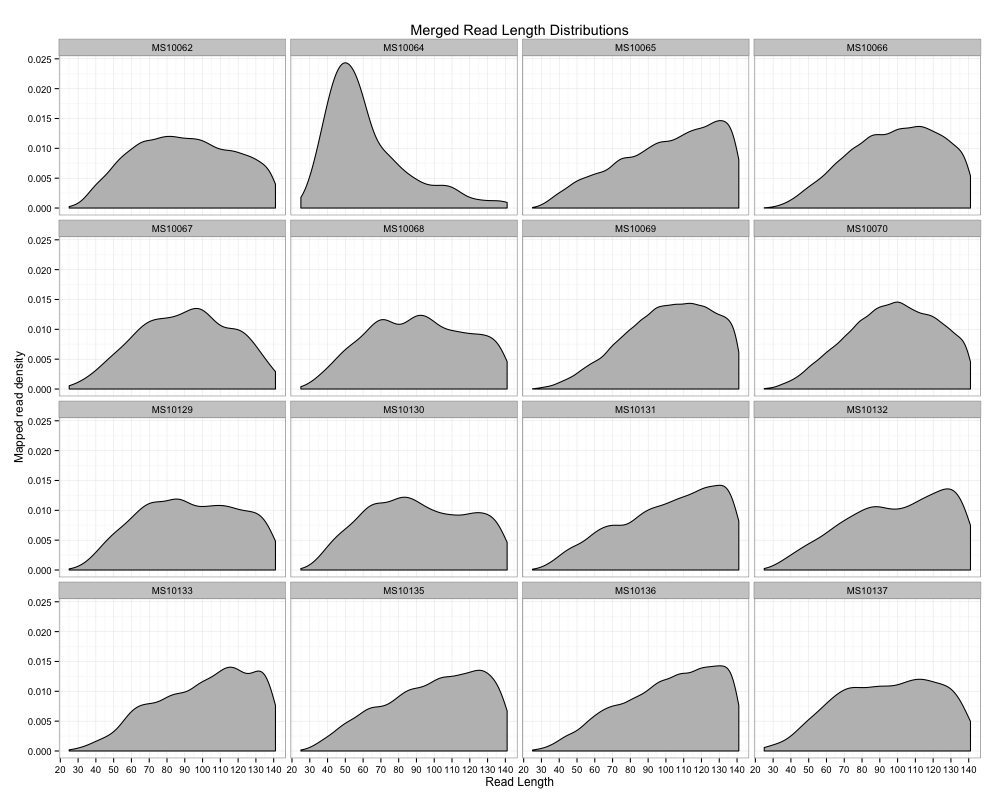

Supplement: S3 Fig — Number of reads and fragment length are provided. (PNG) [file pone.0138536.s003.png]
