# Supplementary material for: Complete Mitochondrial Genomes of New Zealand’s First Dogs
Source: PLoS One. 2015 Oct 7;10(10):e0138536. doi: 10.1371/journal.pone.0138536 (PMC4596854; doi:10.1371/journal.pone.0138536)

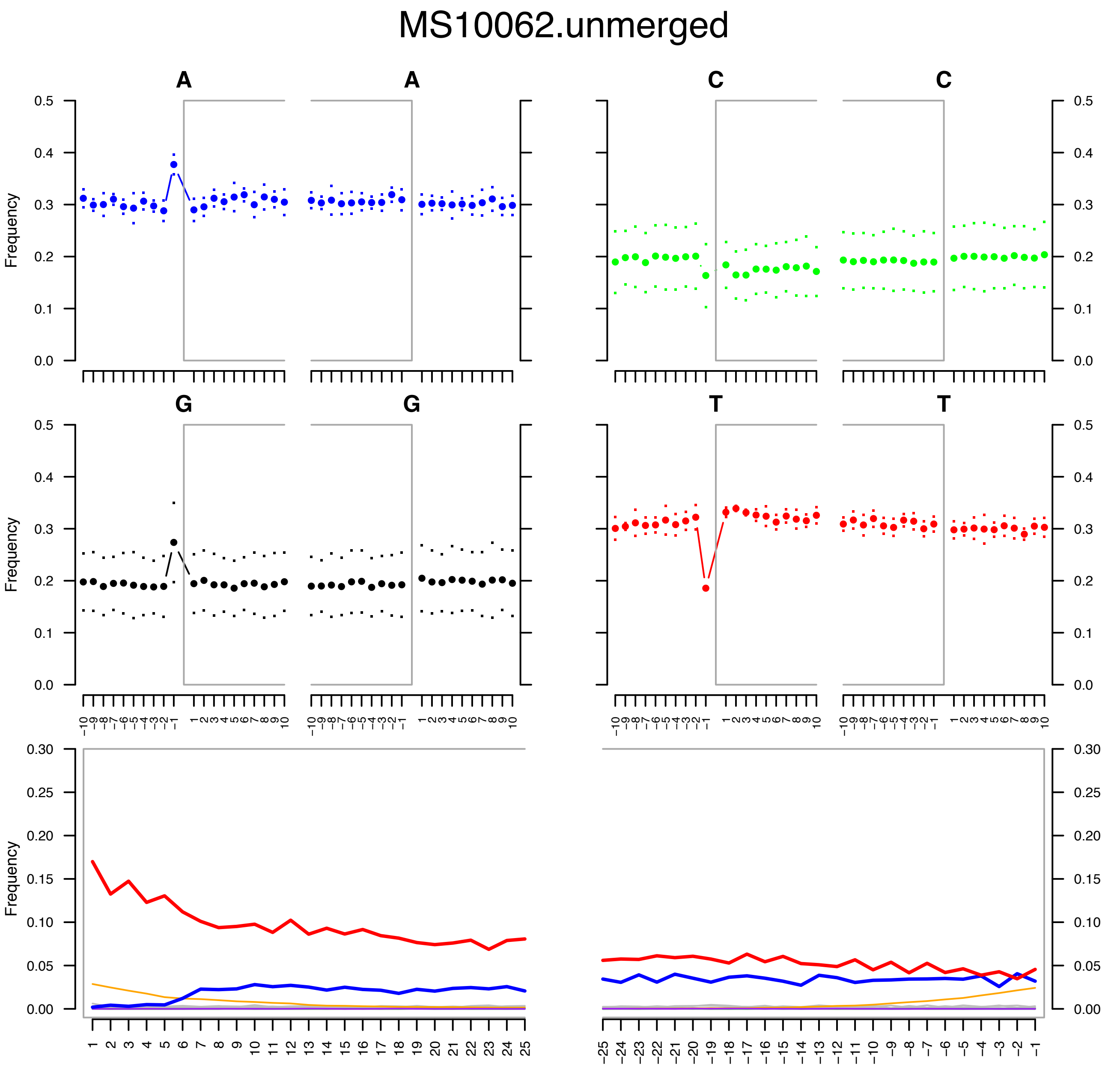
MS10062.unmerged
A
A
C
C
G
G
T
T
Frequency
Frequency
Frequency
0.0
0.1
0.2
0.3
0.4
0.5
0.00
0.05
0.10
0.15
0.20
0.25
0.30

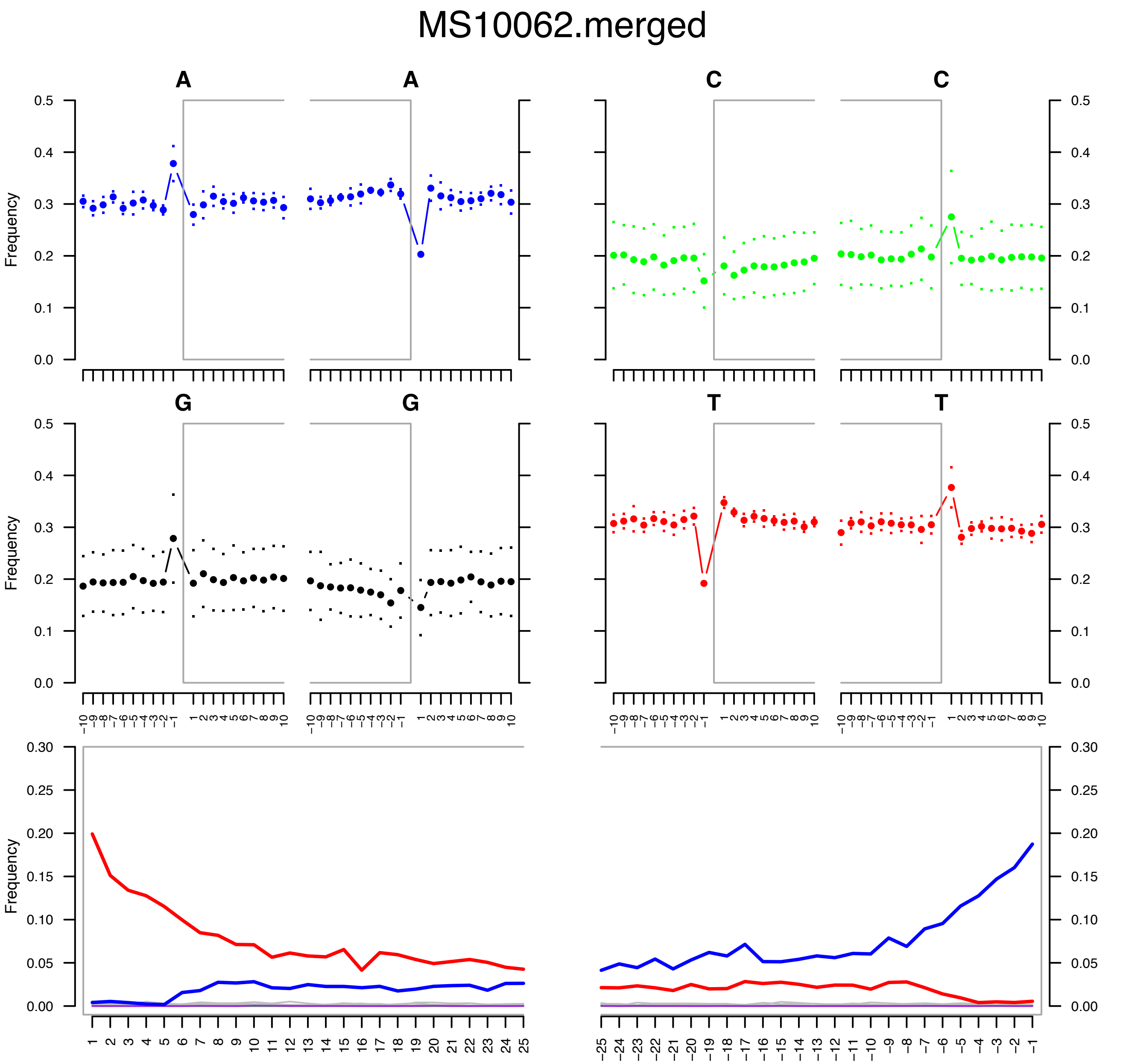
MS10062.merged
A
A
C
C
G
G
T
T
Frequency
Frequency
Frequency

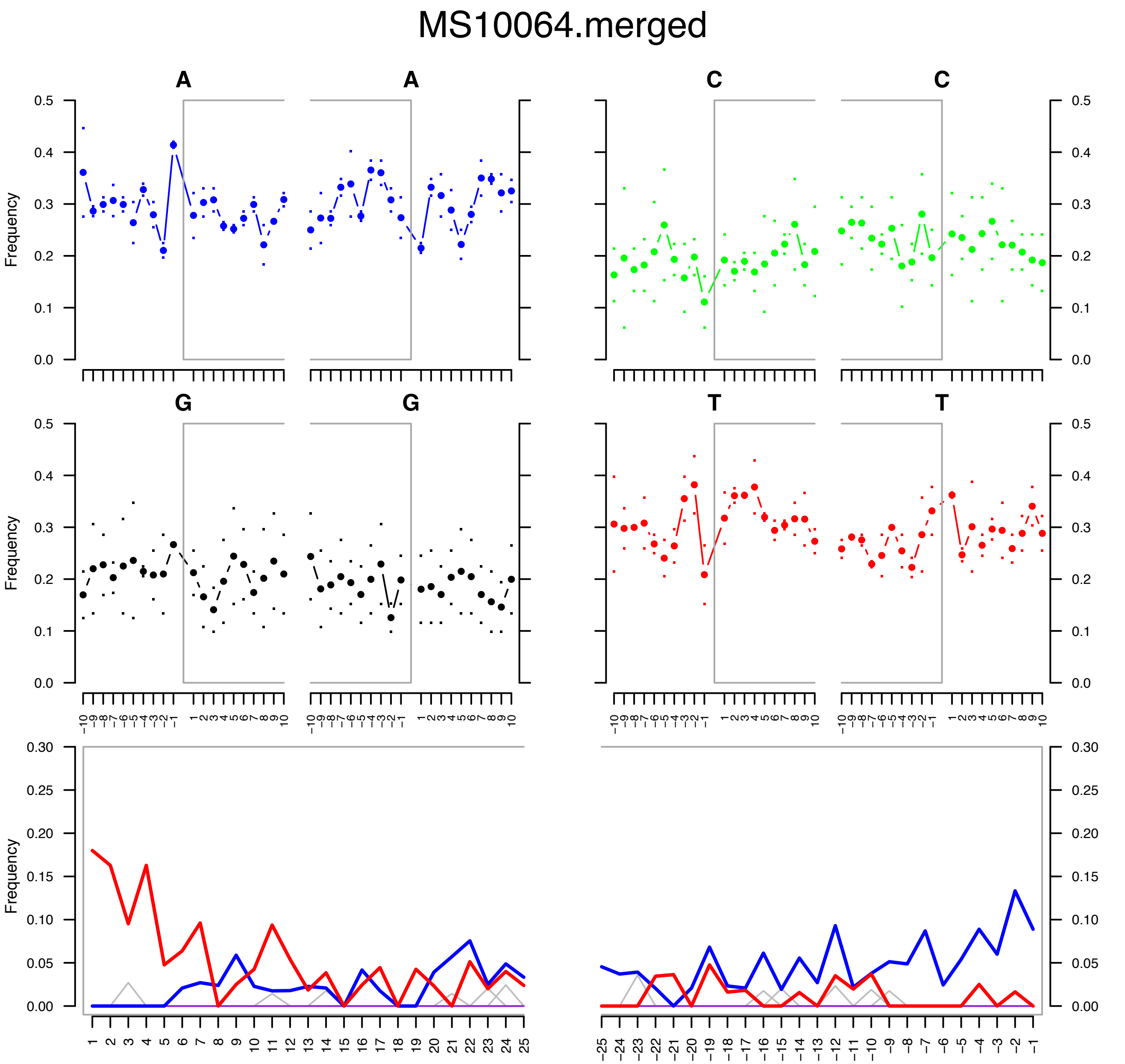

MS10064.merged
A
A
C
C
G
G
T
T
Frequency

MS10065.unmerged

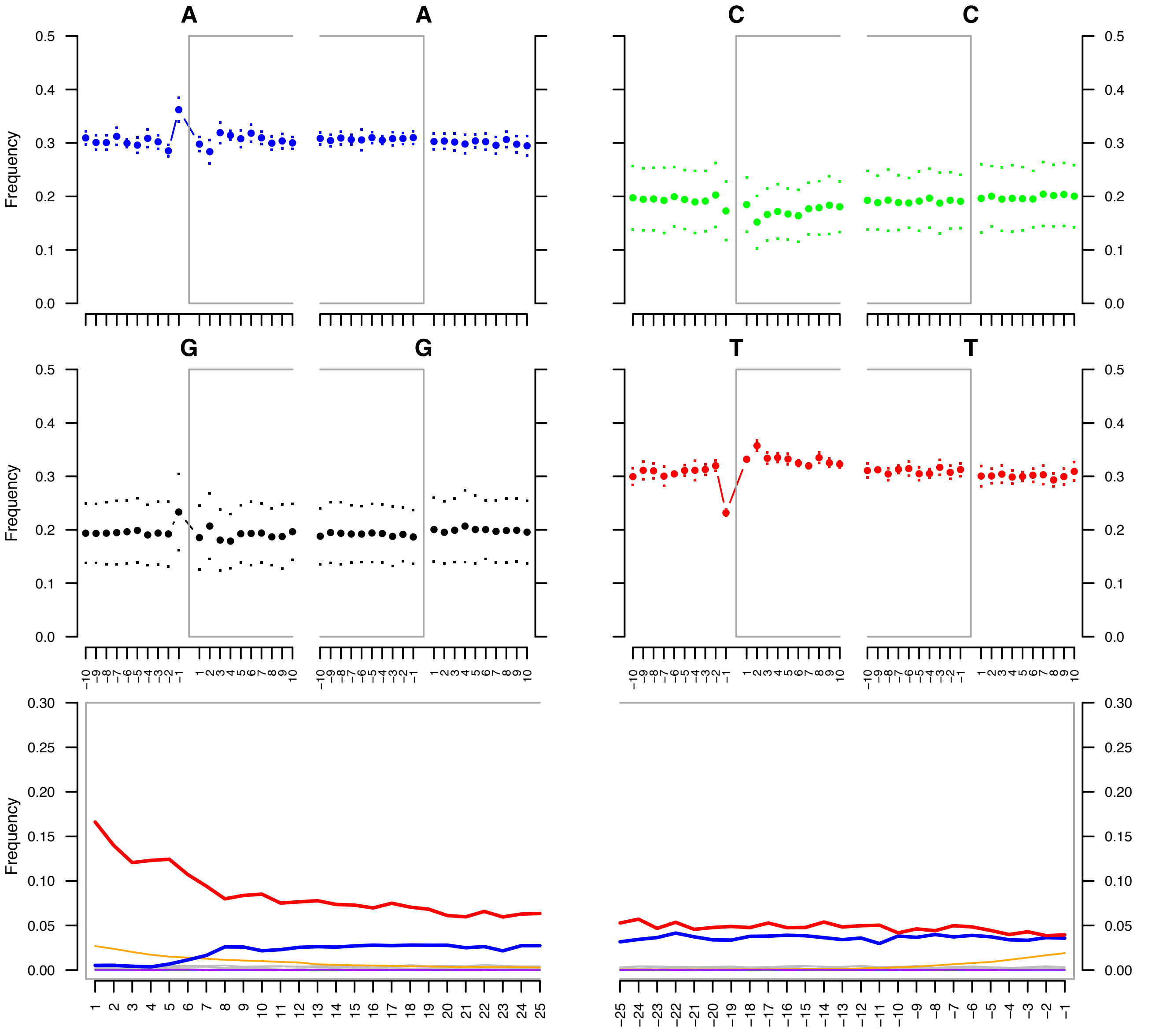

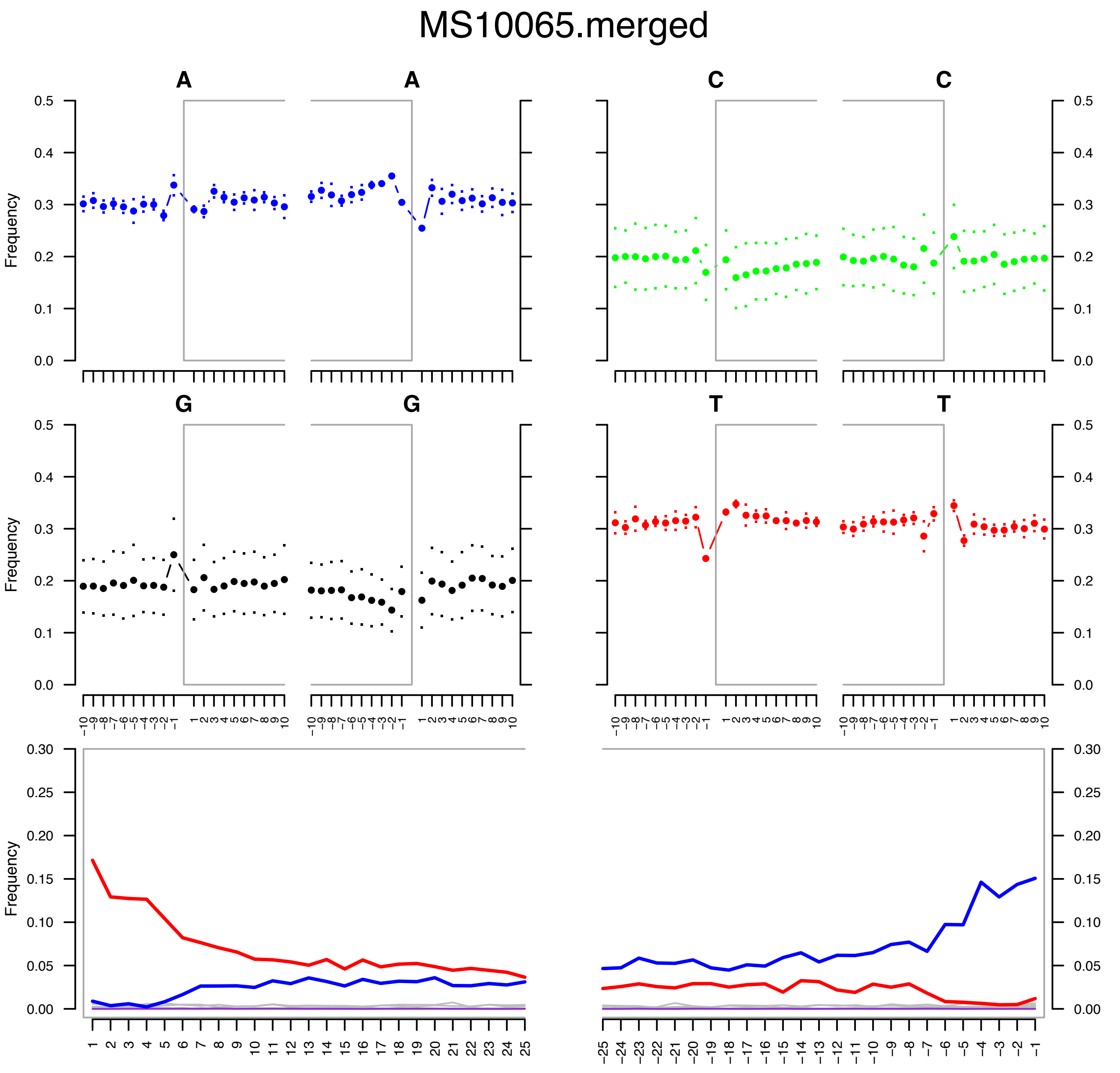
MS10065.merged
A
A
C
C
G
G
T
T
Frequency
Frequency
Frequency

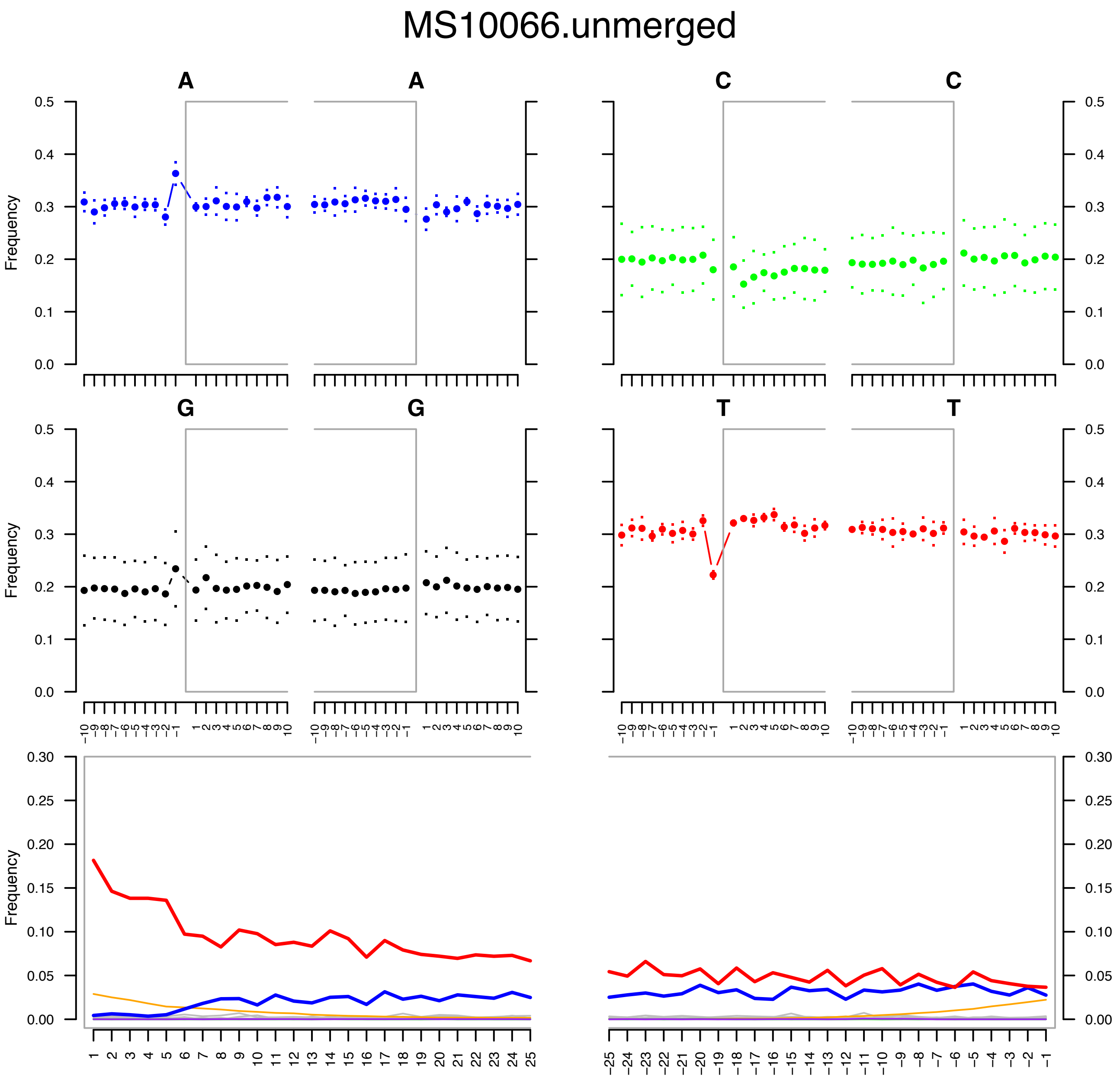
MS10066.unmerged
A
A
C
C
G
G
T
T
Frequency
Frequency
Frequency

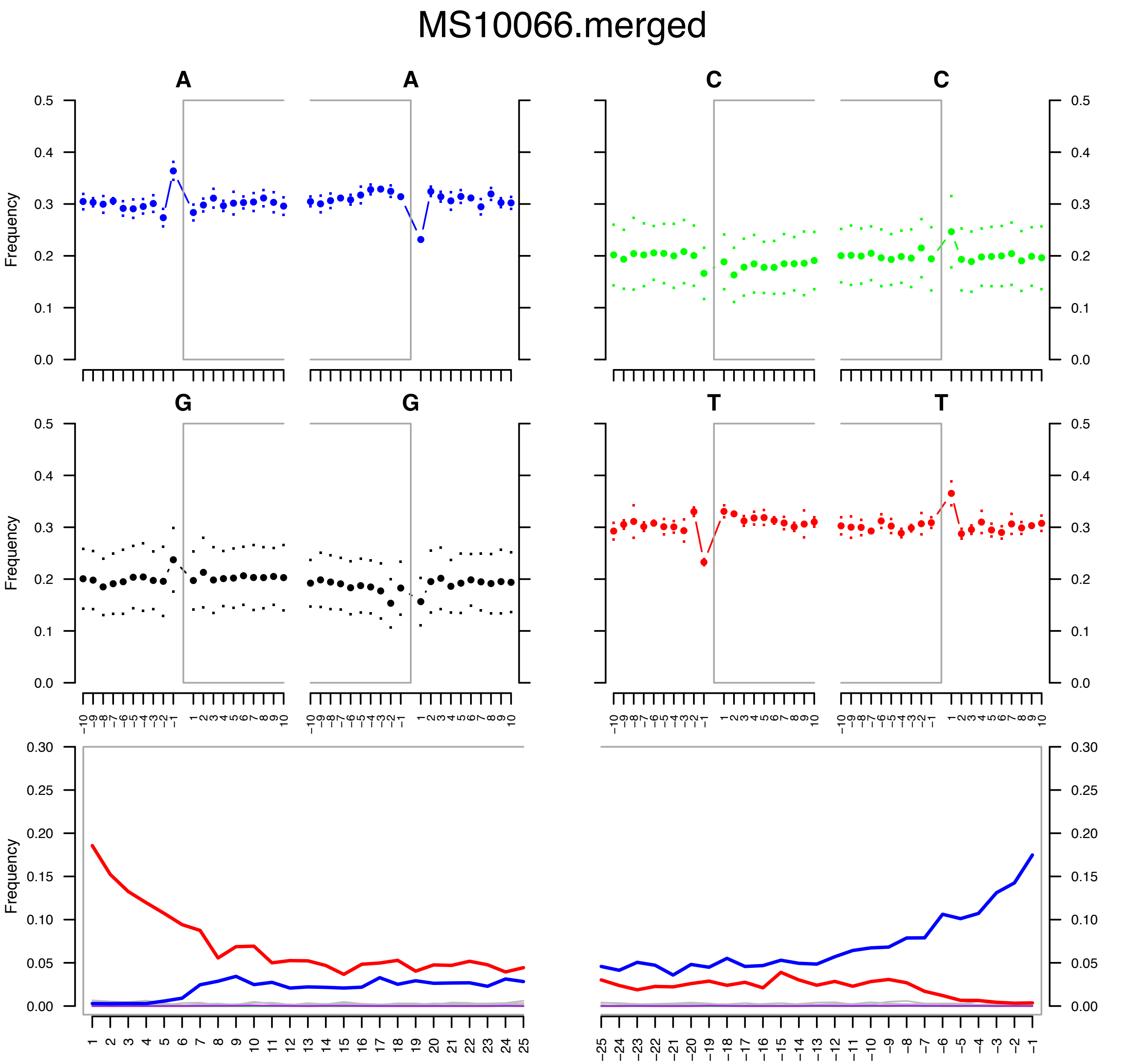
MS10066.merged
A
A
C
C
G
G
T
T
Frequency
Frequency
Frequency

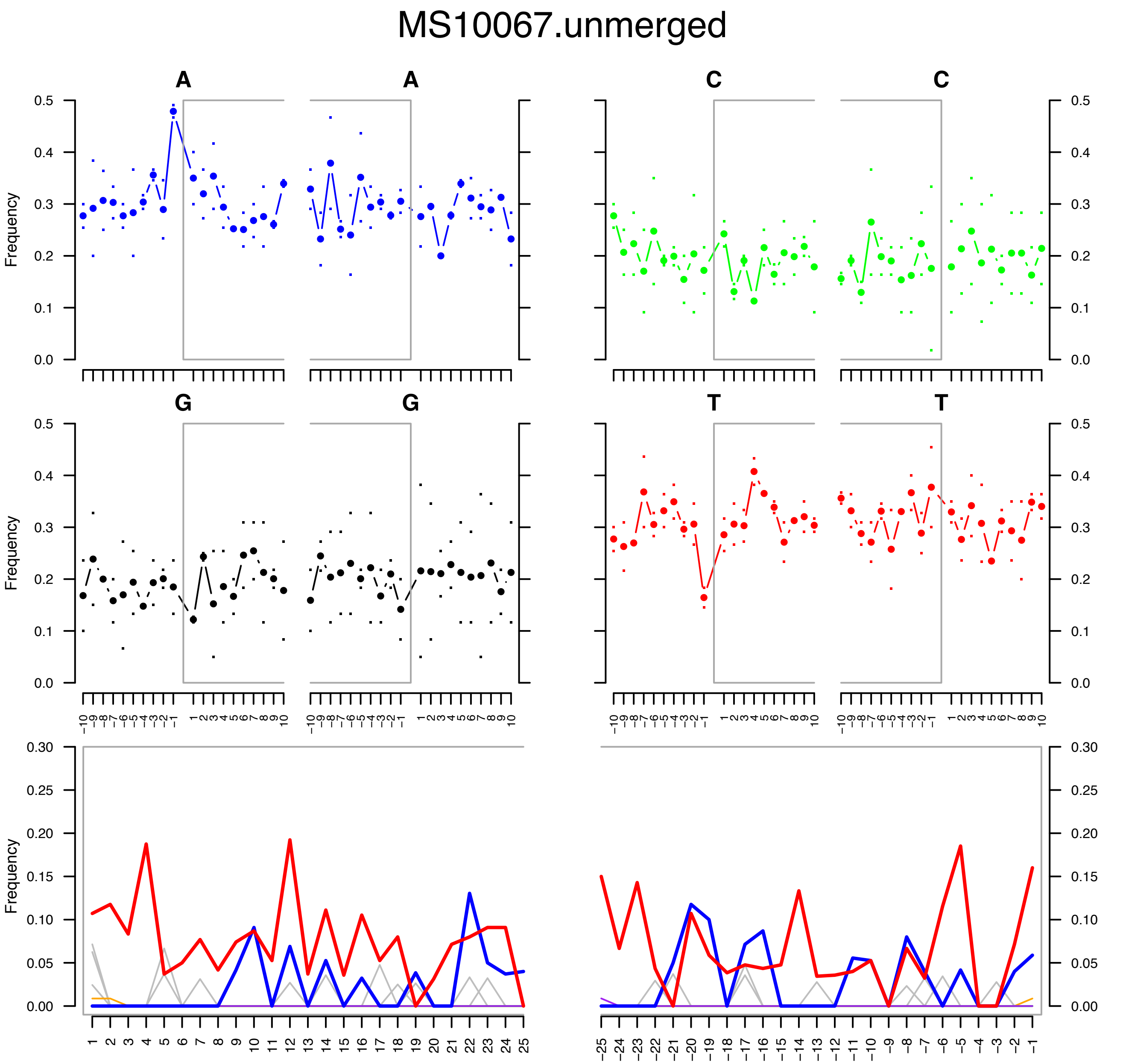

MS10067.unmerged
A
A
C
C
G
G
T
T
Frequency
Frequency
Frequency

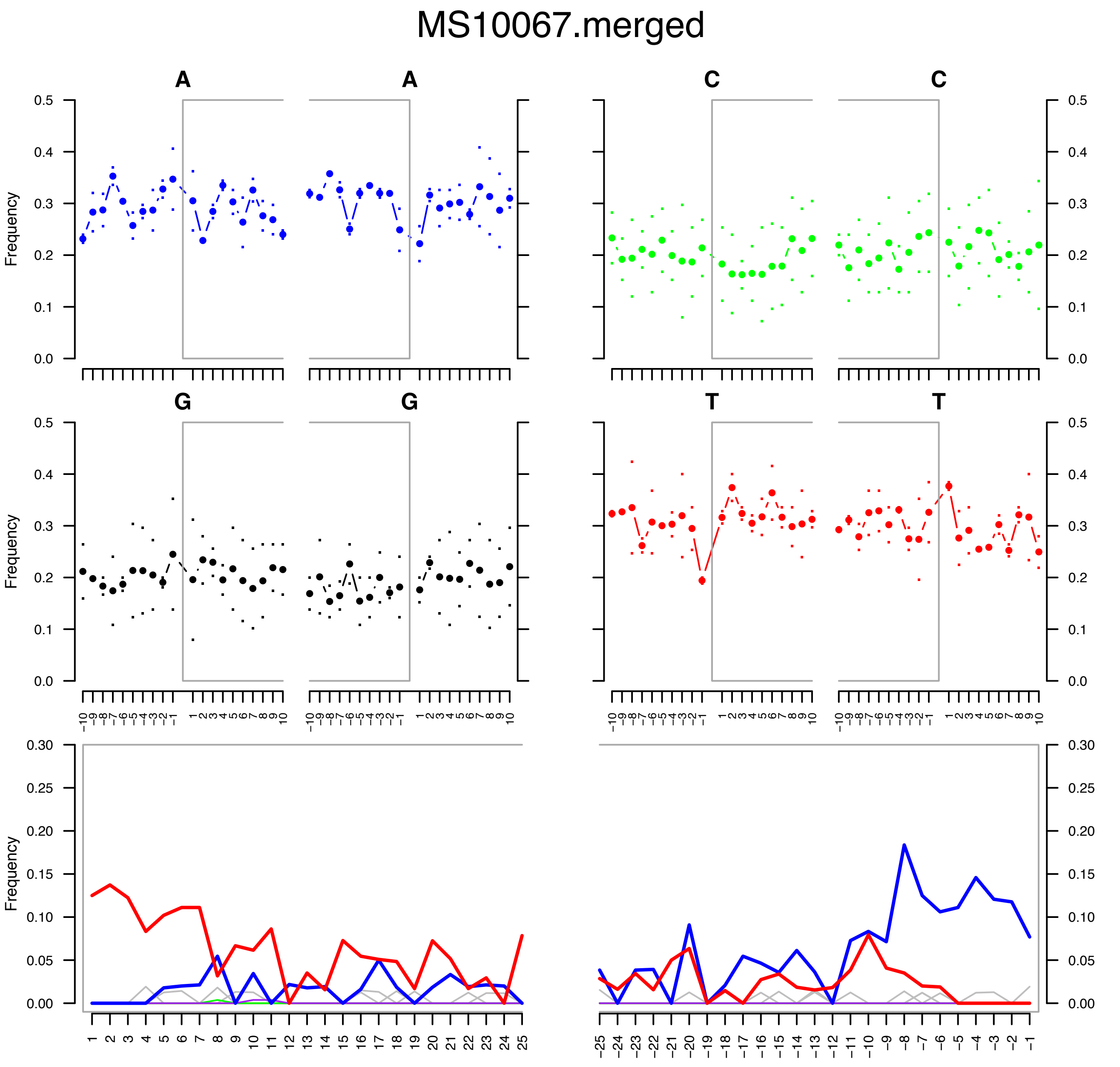
MS10067.merged
A
A
C
C
G
G
T
T
Frequency
Frequency
Frequency

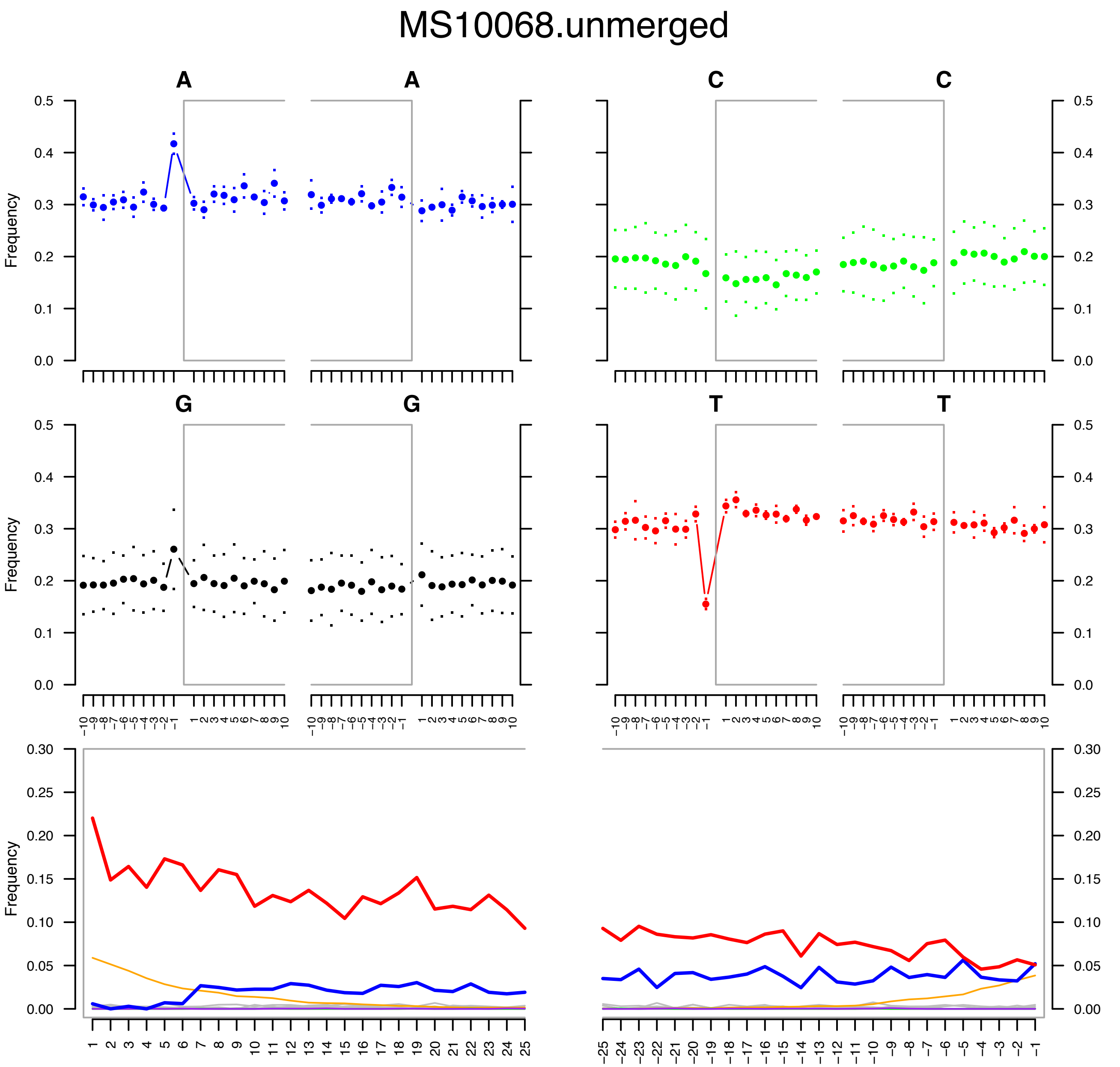

MS10068.unmerged
A
A
C
C
G
G
T
T
Frequency
Frequency
Frequency

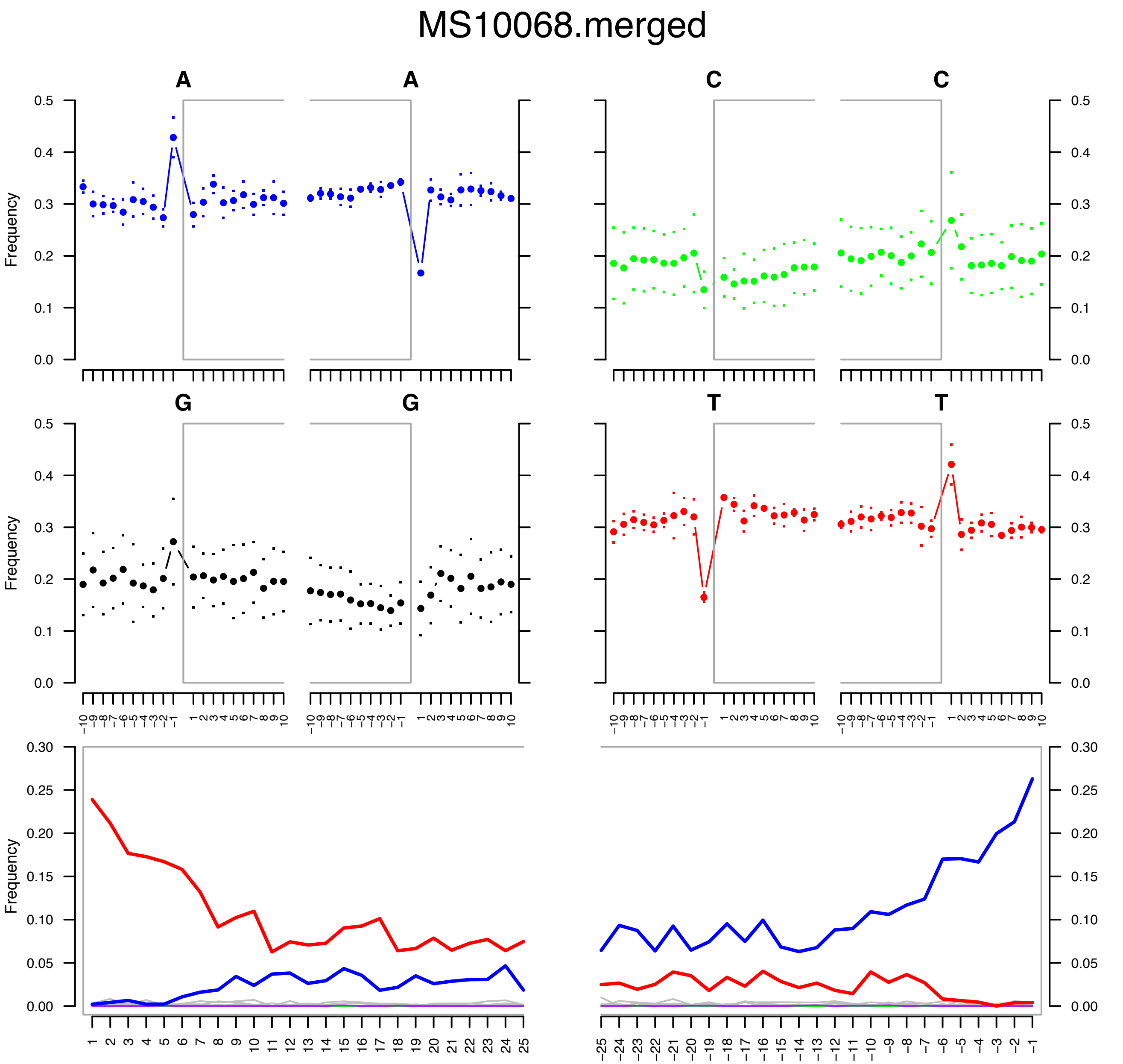
MS10068.merged
A
A
C
C
G
G
T
T
Frequency
Frequency
Frequency

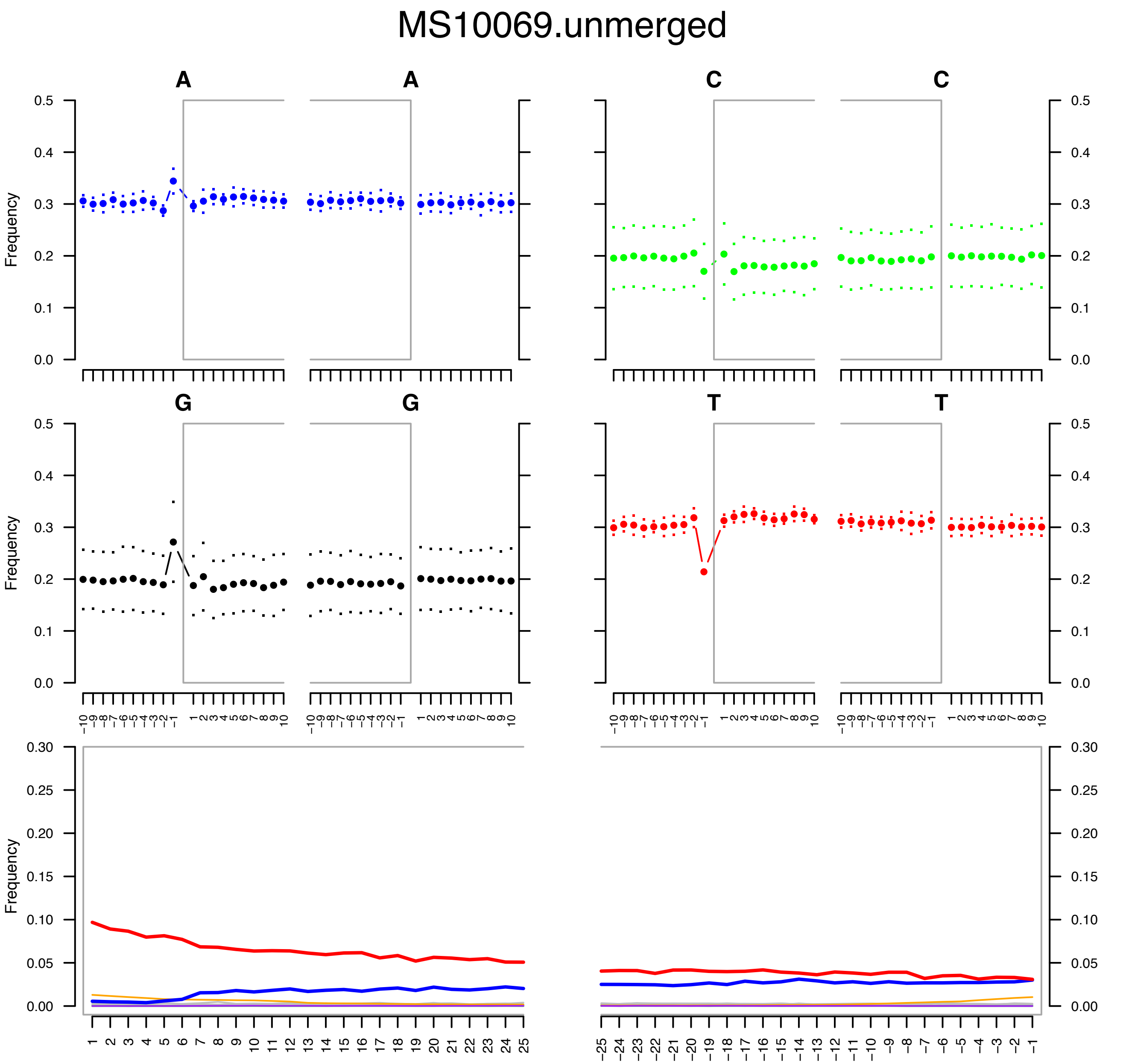
MS10069.unmerged
A
A
C
C
G
G
T
T
Frequency
Frequency
Frequency

MS10069.merged

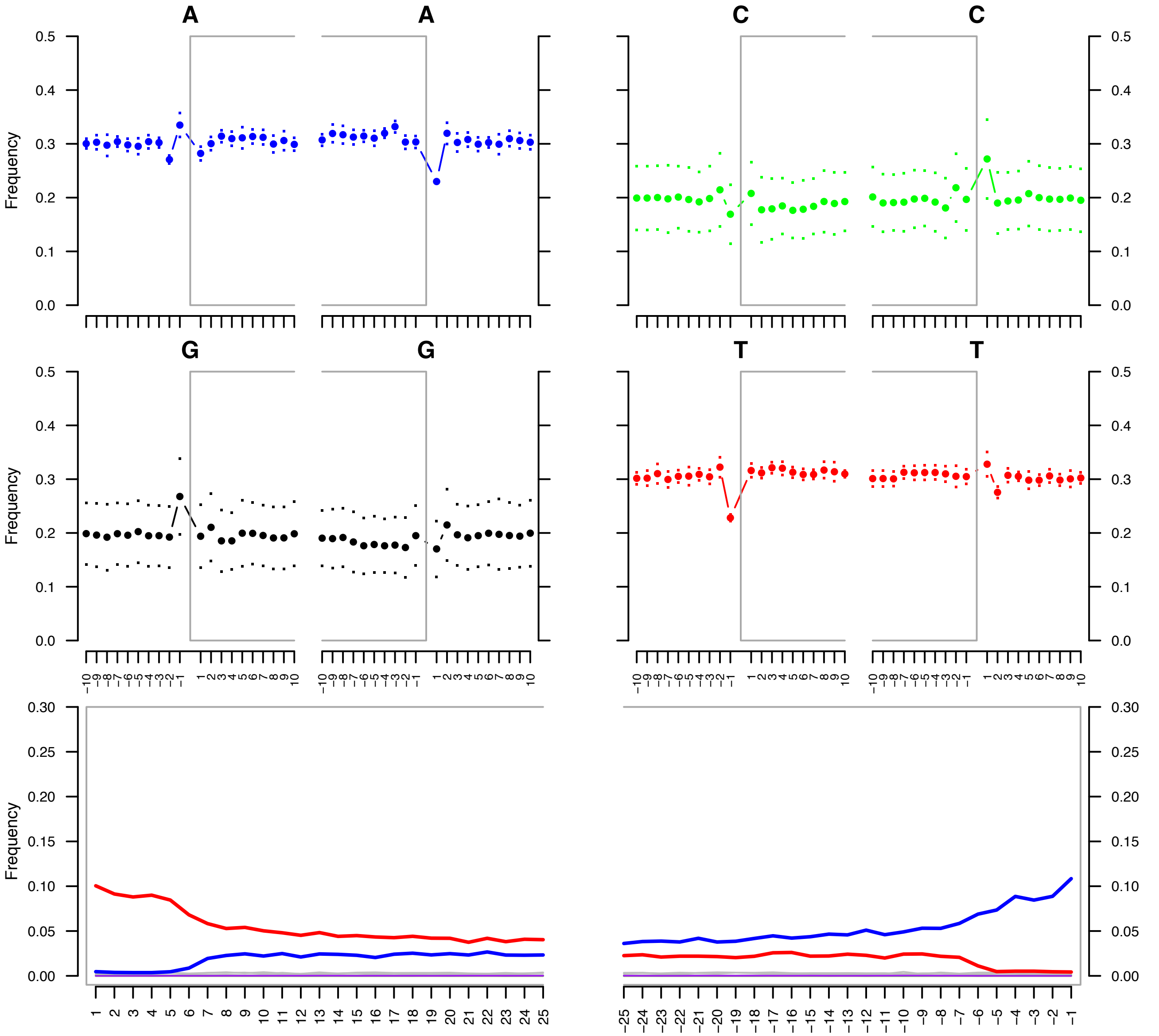

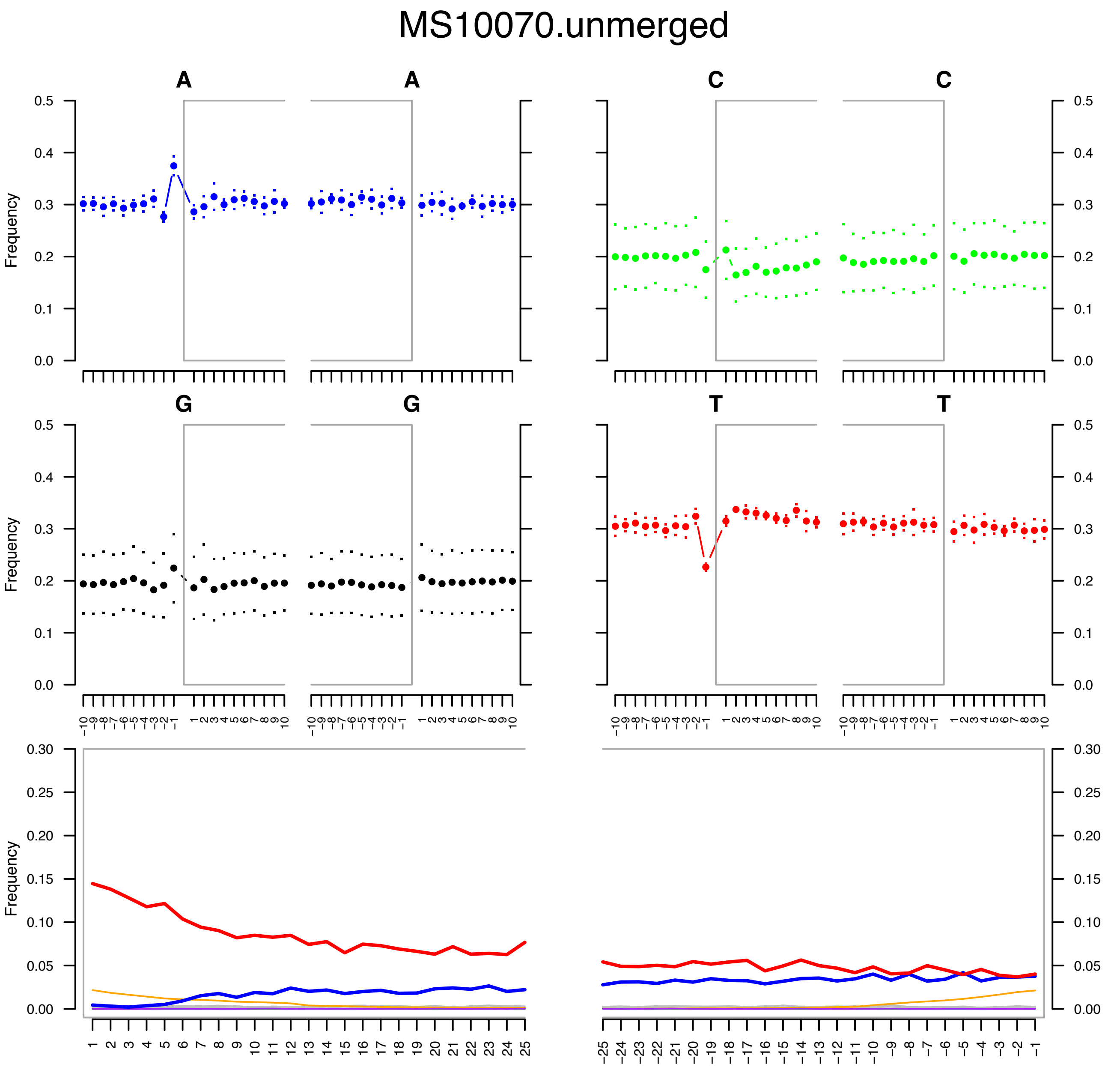
MS10070.unmerged
A
A
C
C
G
G
T
T
Frequency
Frequency
Frequency

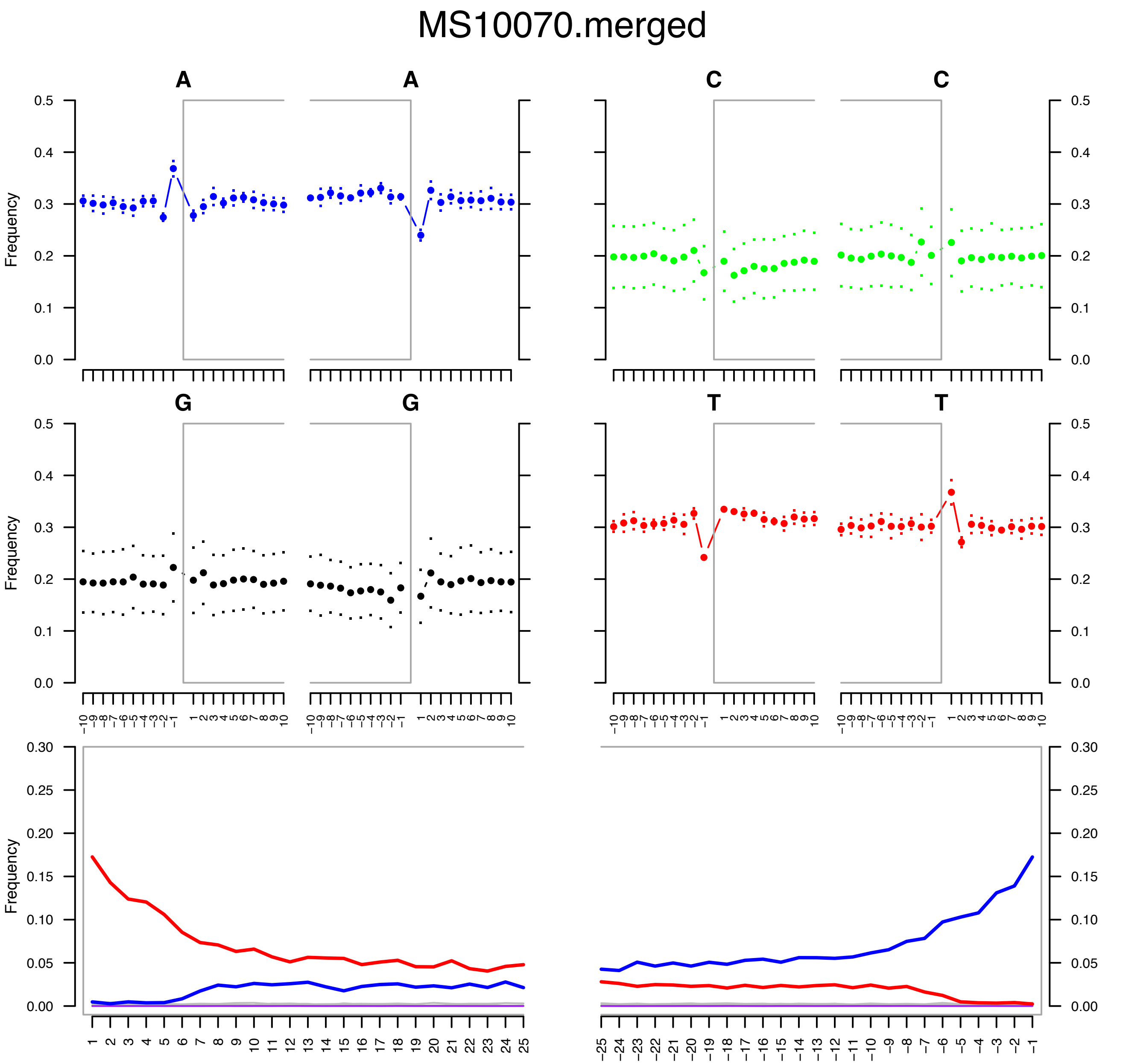
MS10070.merged
A
A
C
C
G
G
T
T
Frequency
Frequency
Frequency

MS10129.unmerged

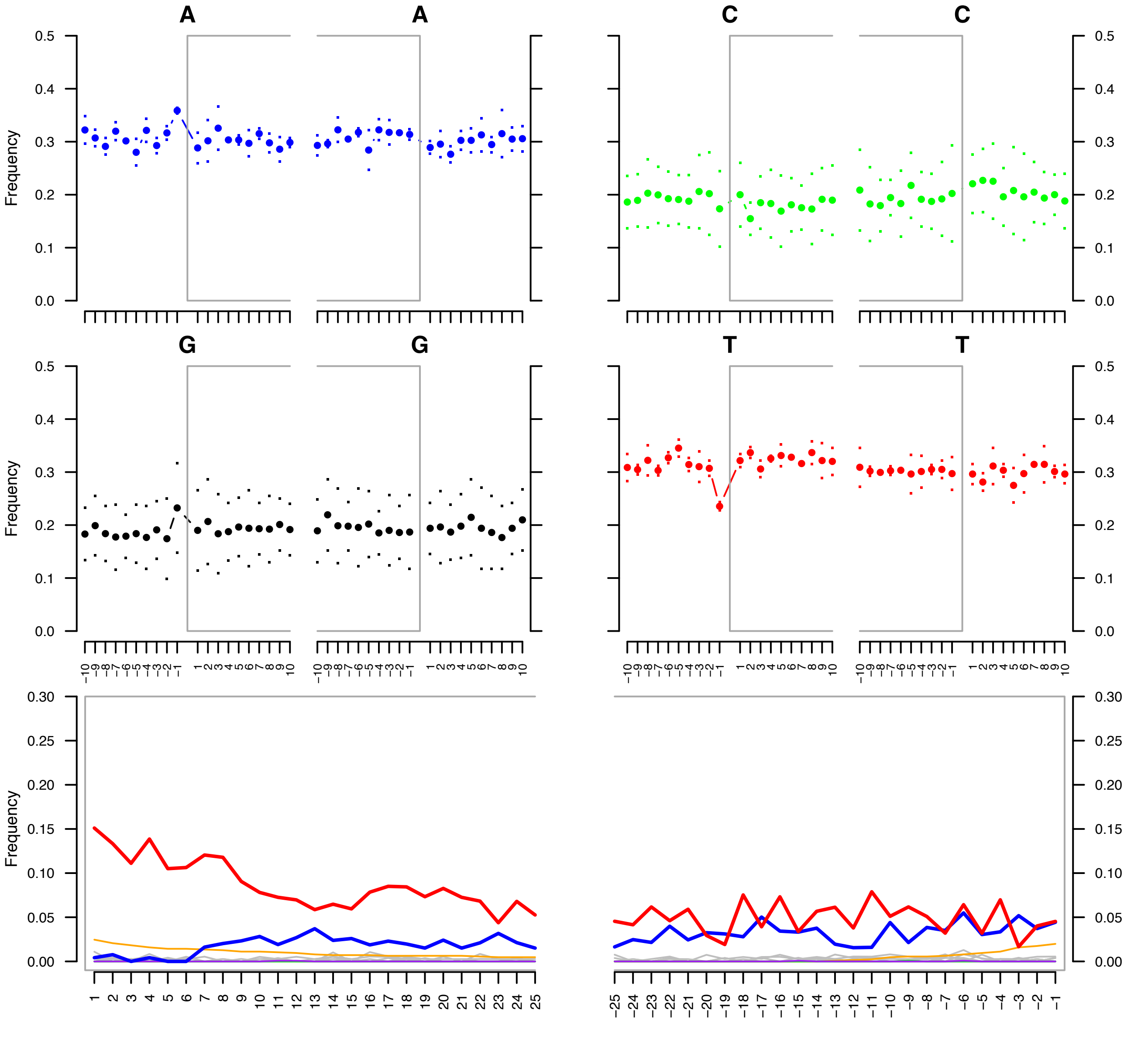

MS10129.merged

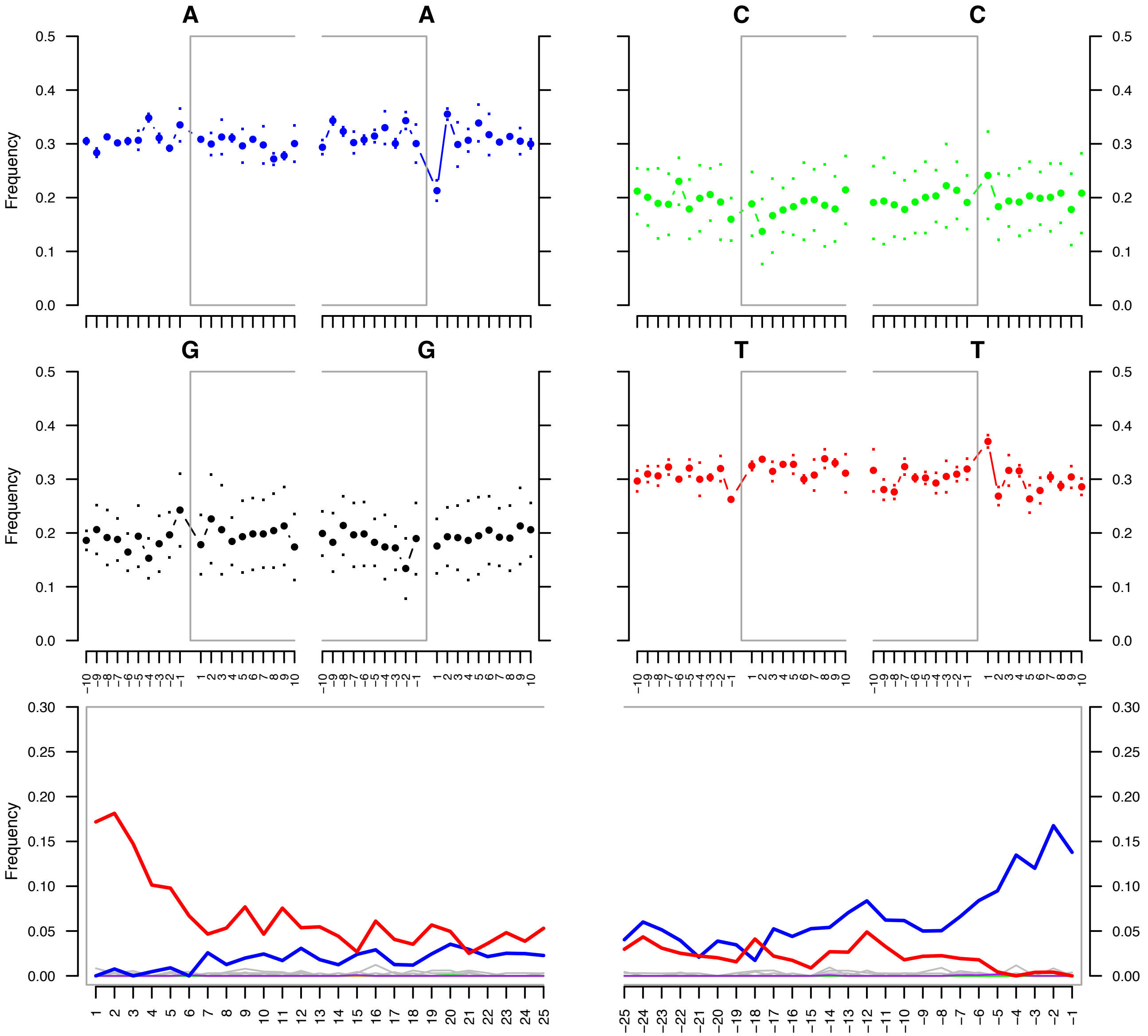

MS10130.unmerged

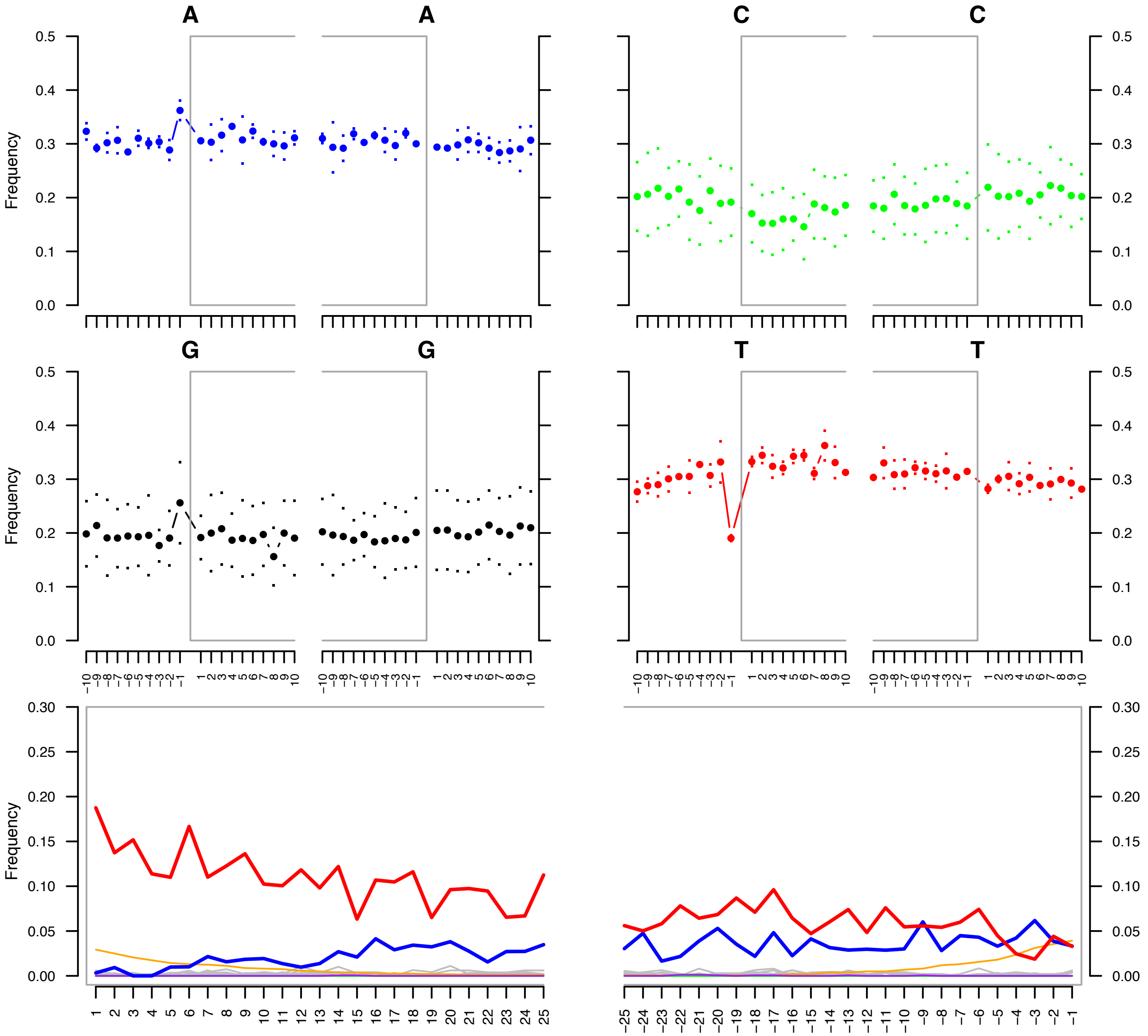

MS10130.merged

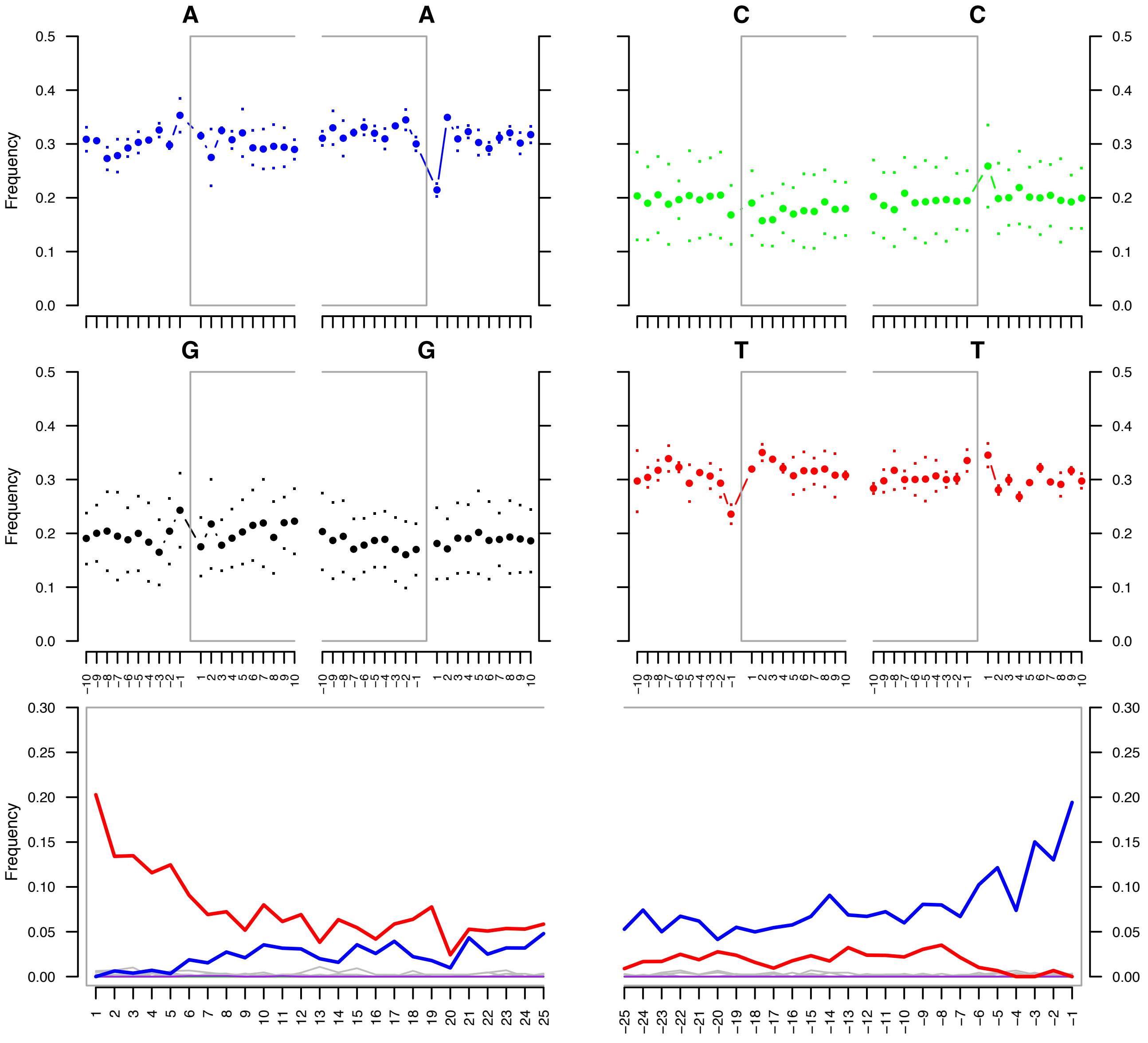

MS10131.unmerged

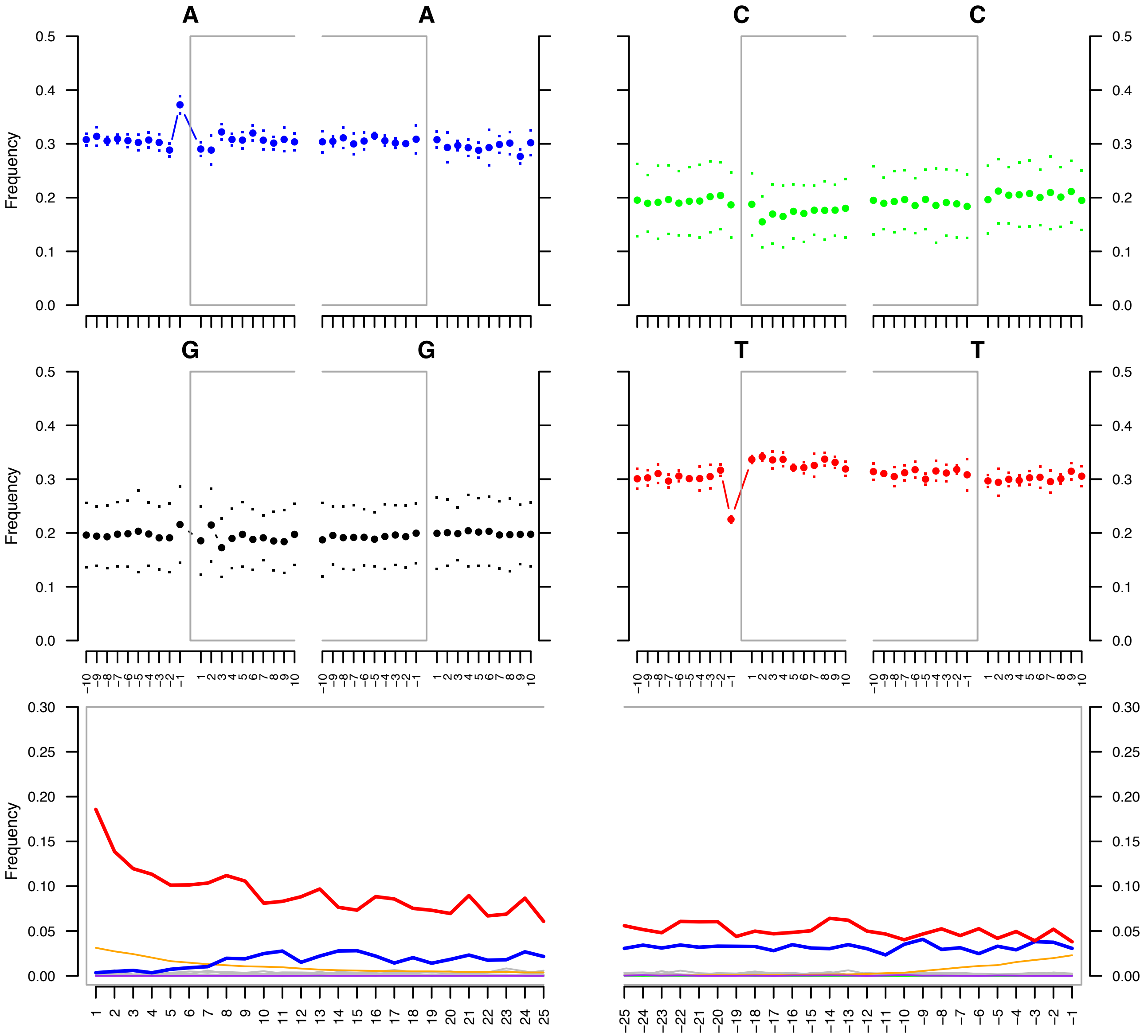

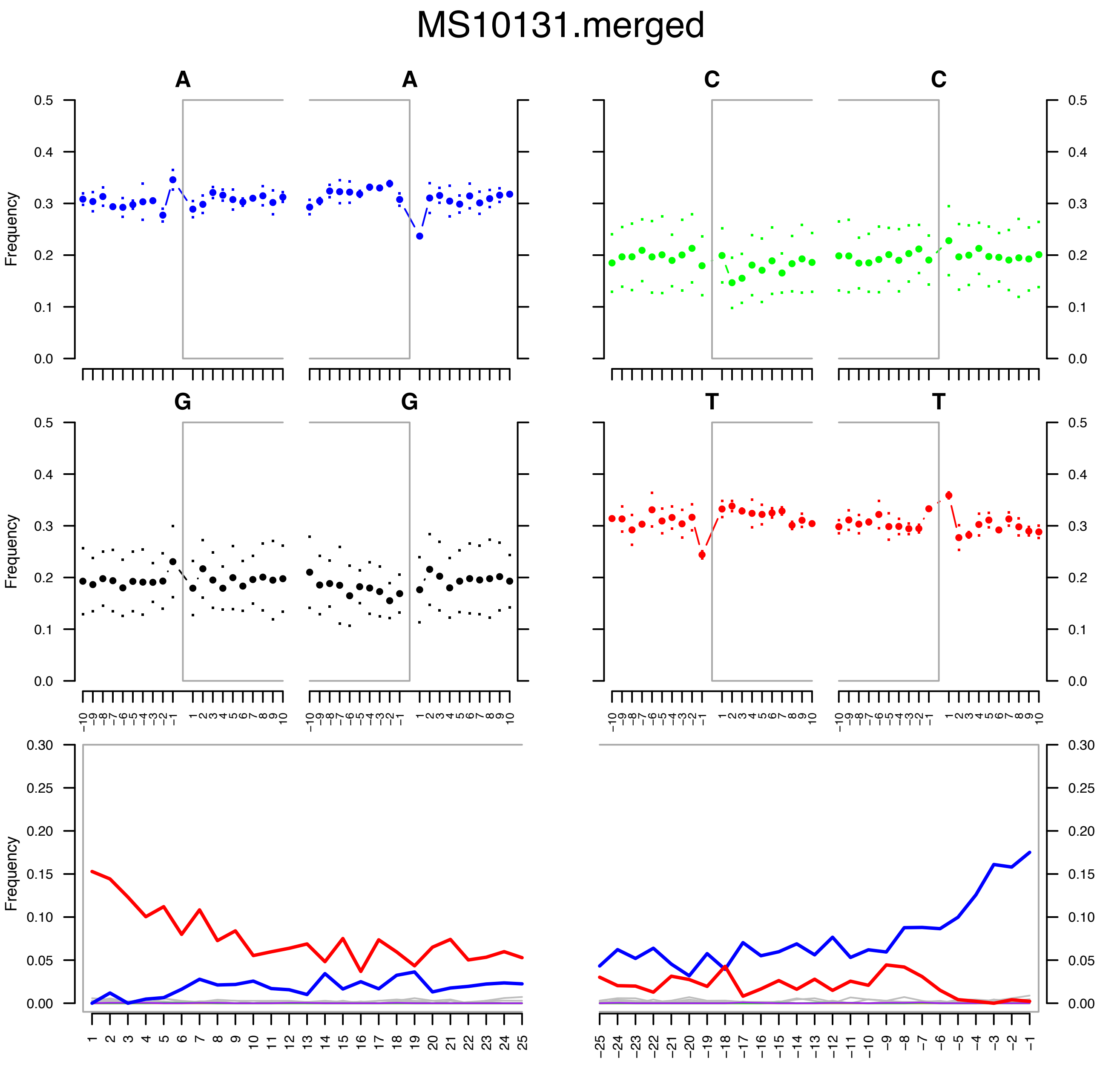
MS10131.merged
A
A
C
C
G
G
T
T
Frequency
Frequency
Frequency

MS10132.unmerged

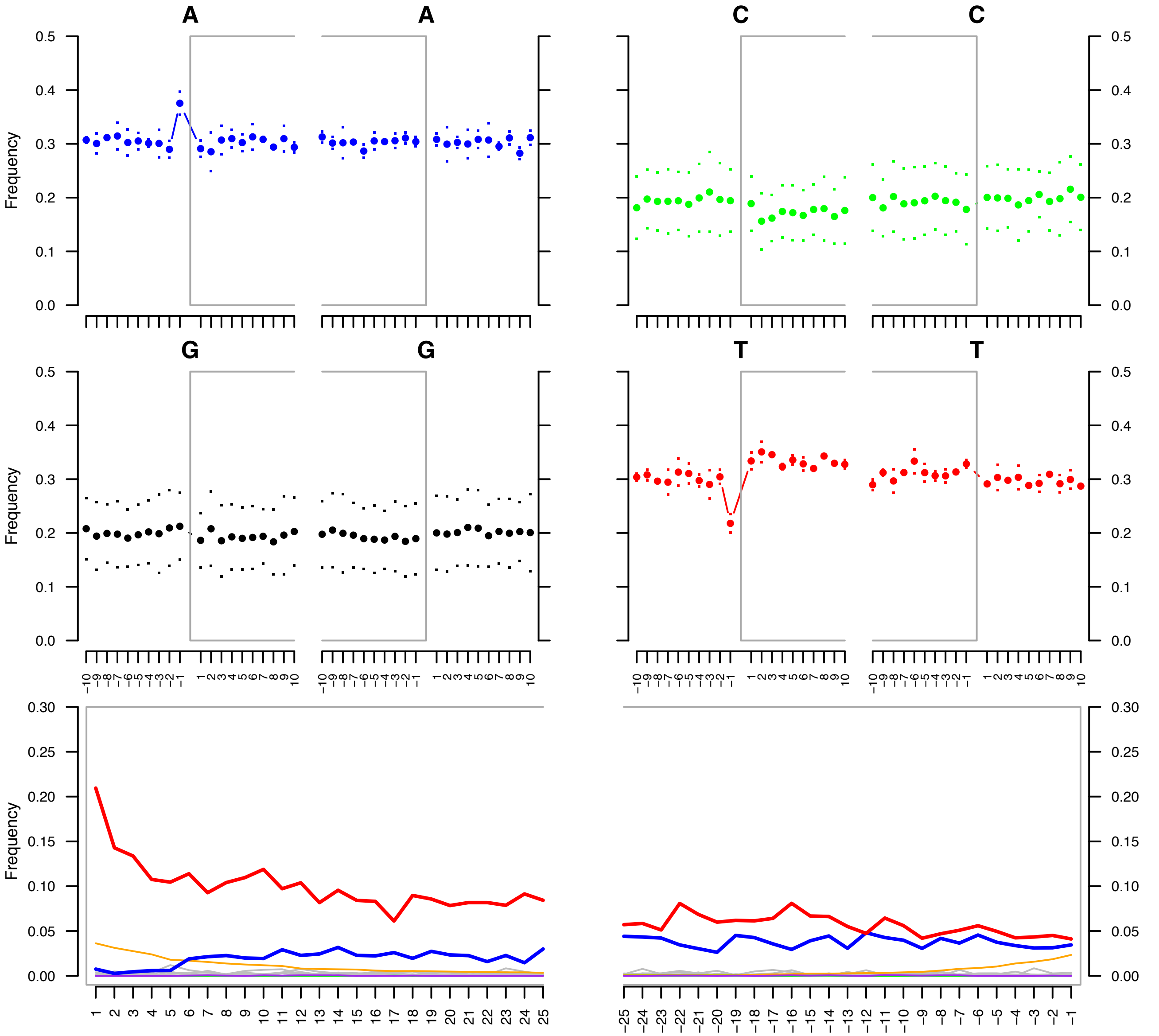

MS10132.merged

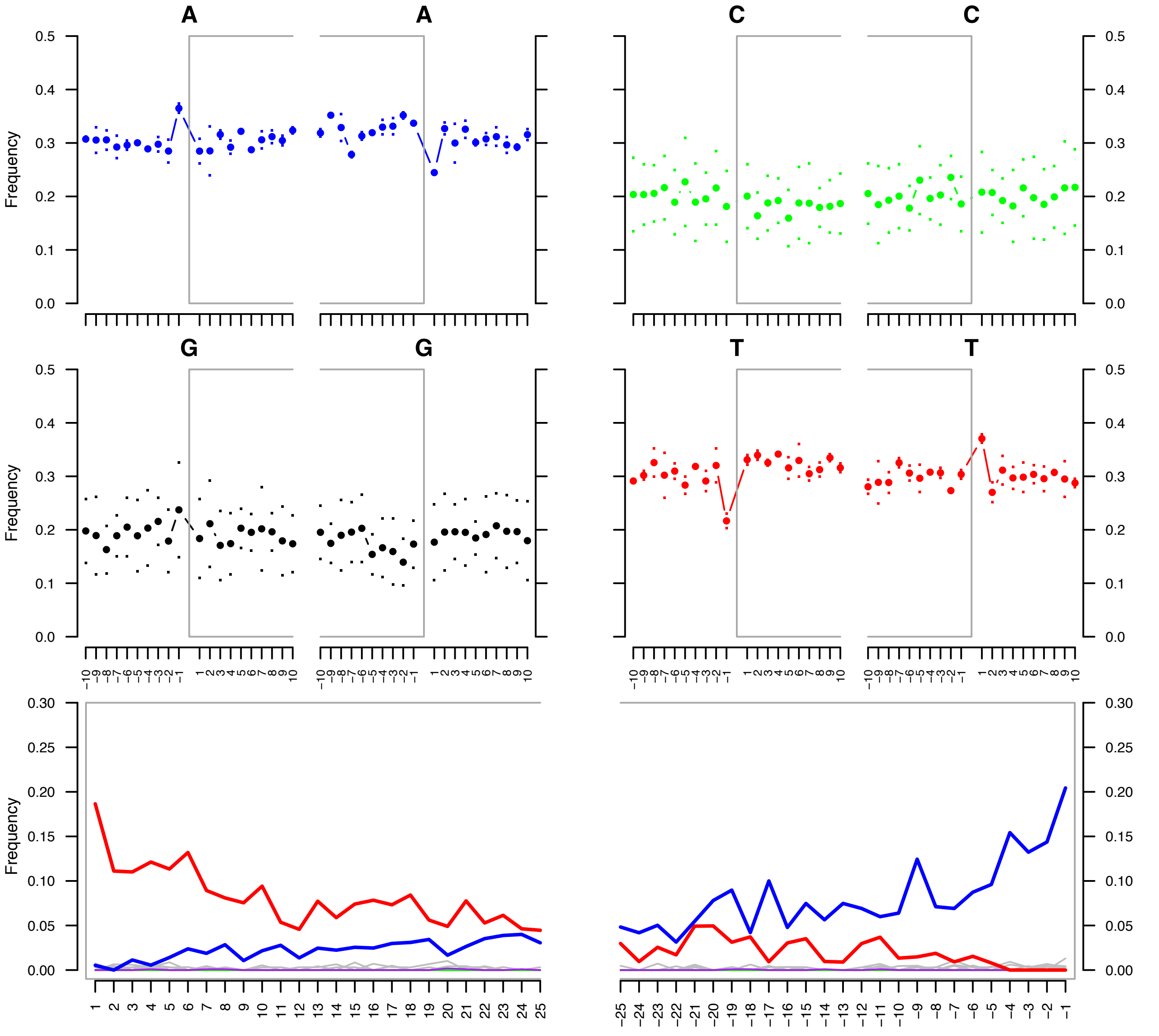

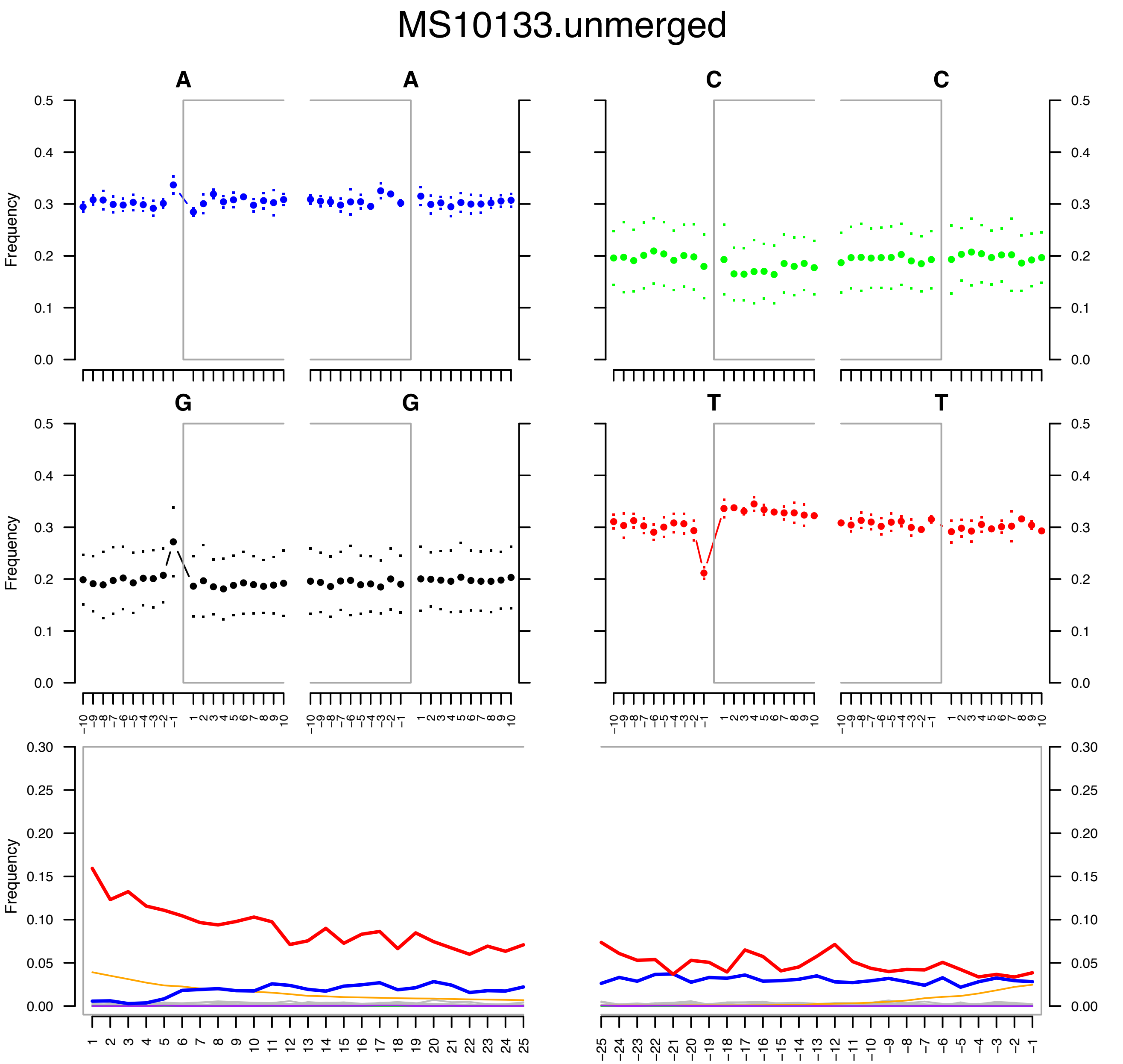

MS10133.unmerged
A
A
C
C
G
G
T
T
Frequency
Frequency
Frequency

MS10133.merged

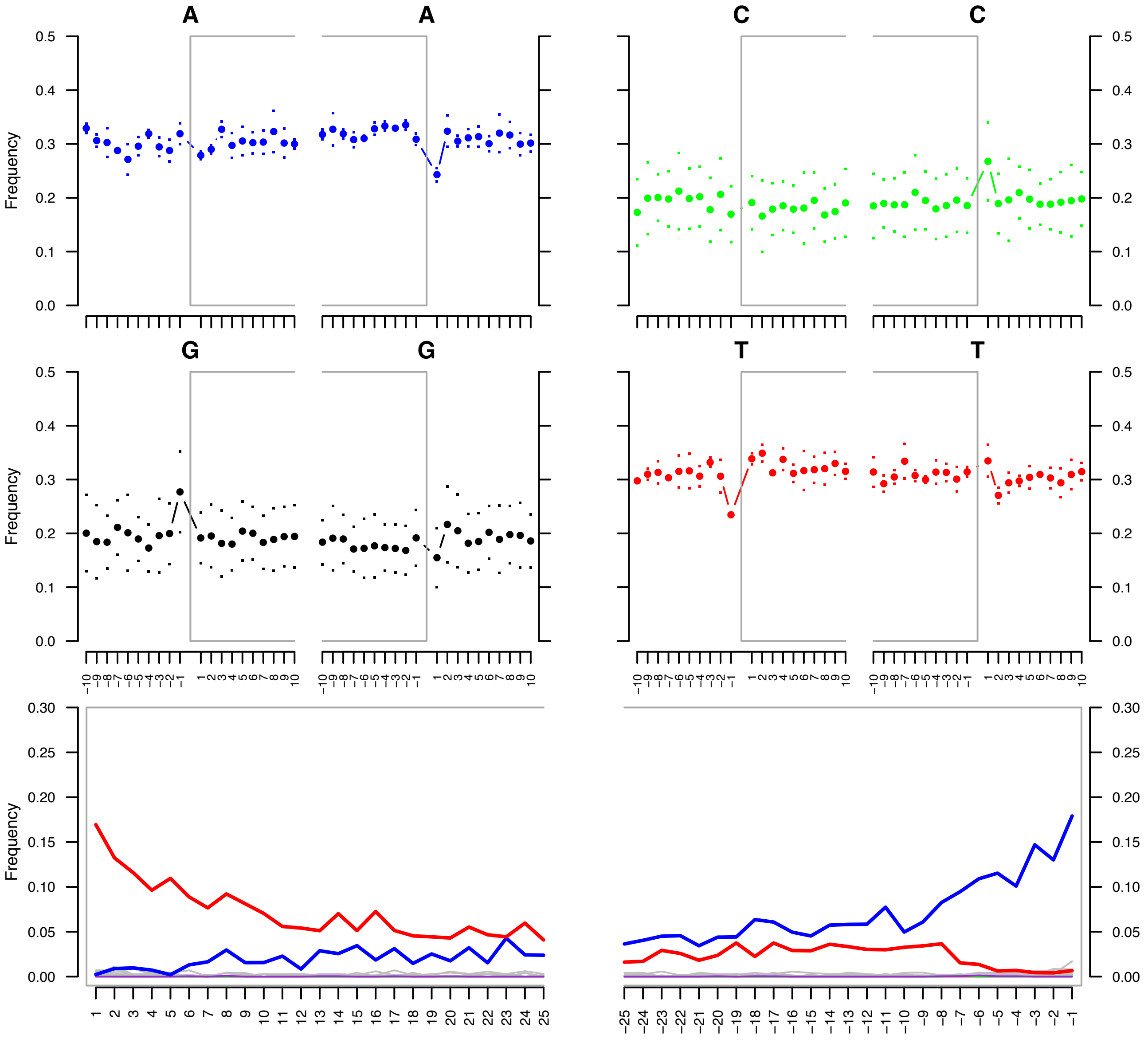

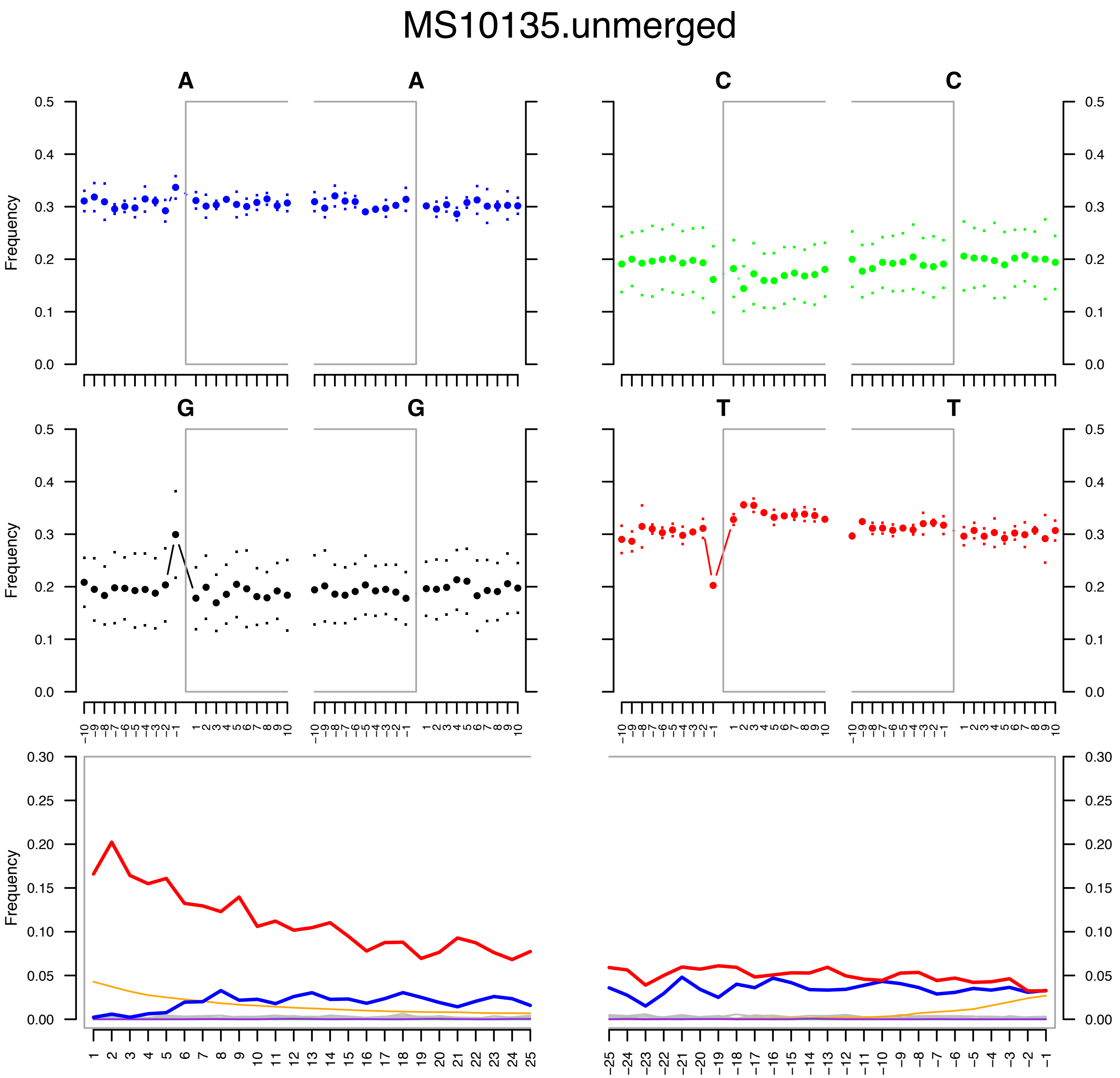

MS10135.unmerged
A
A
C
C
G
G
T
T
Frequency
Frequency
Frequency

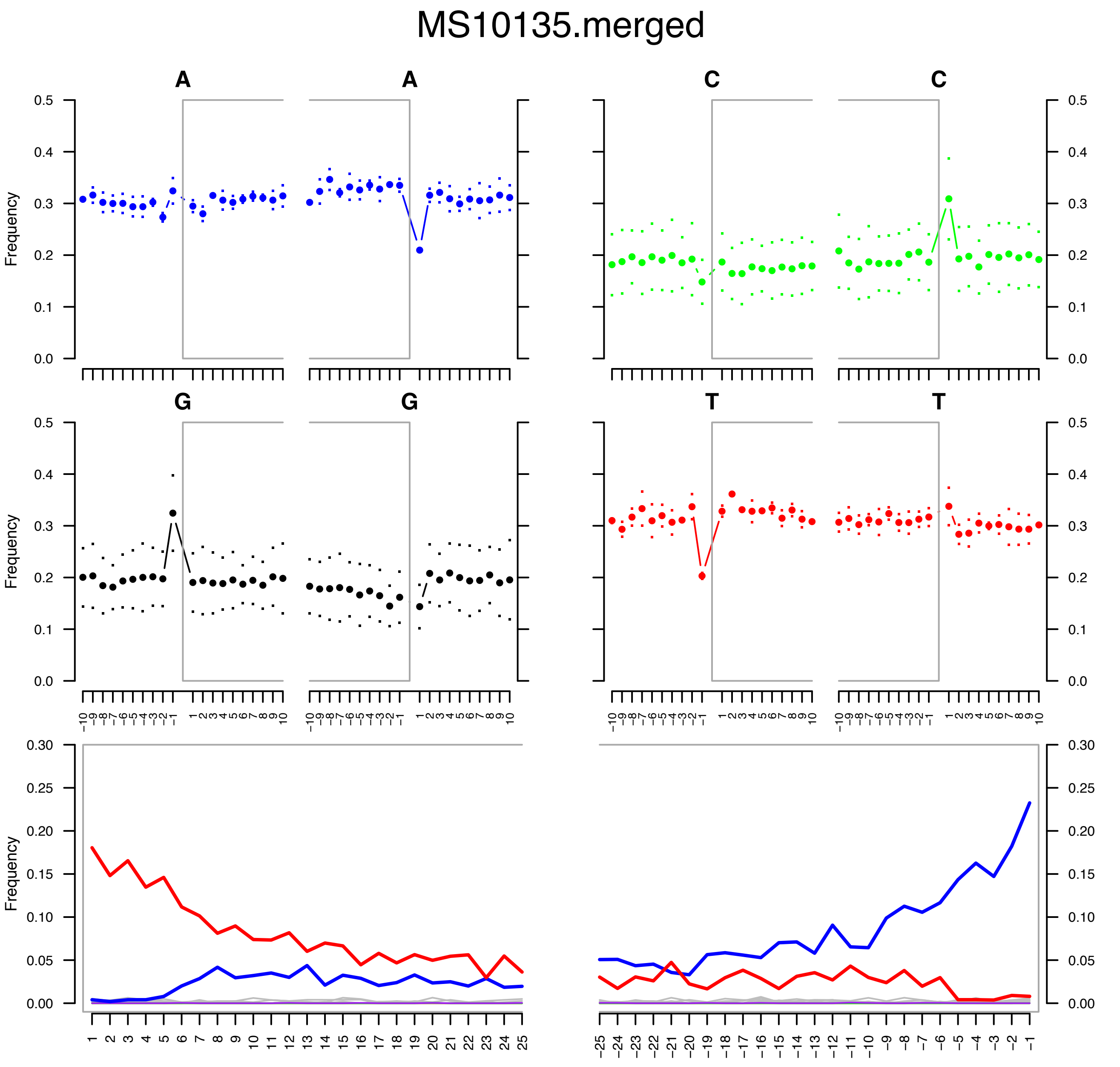
MS10135.merged
A
A
C
C
G
G
T
T
Frequency
Frequency
Frequency

MS10136.unmerged

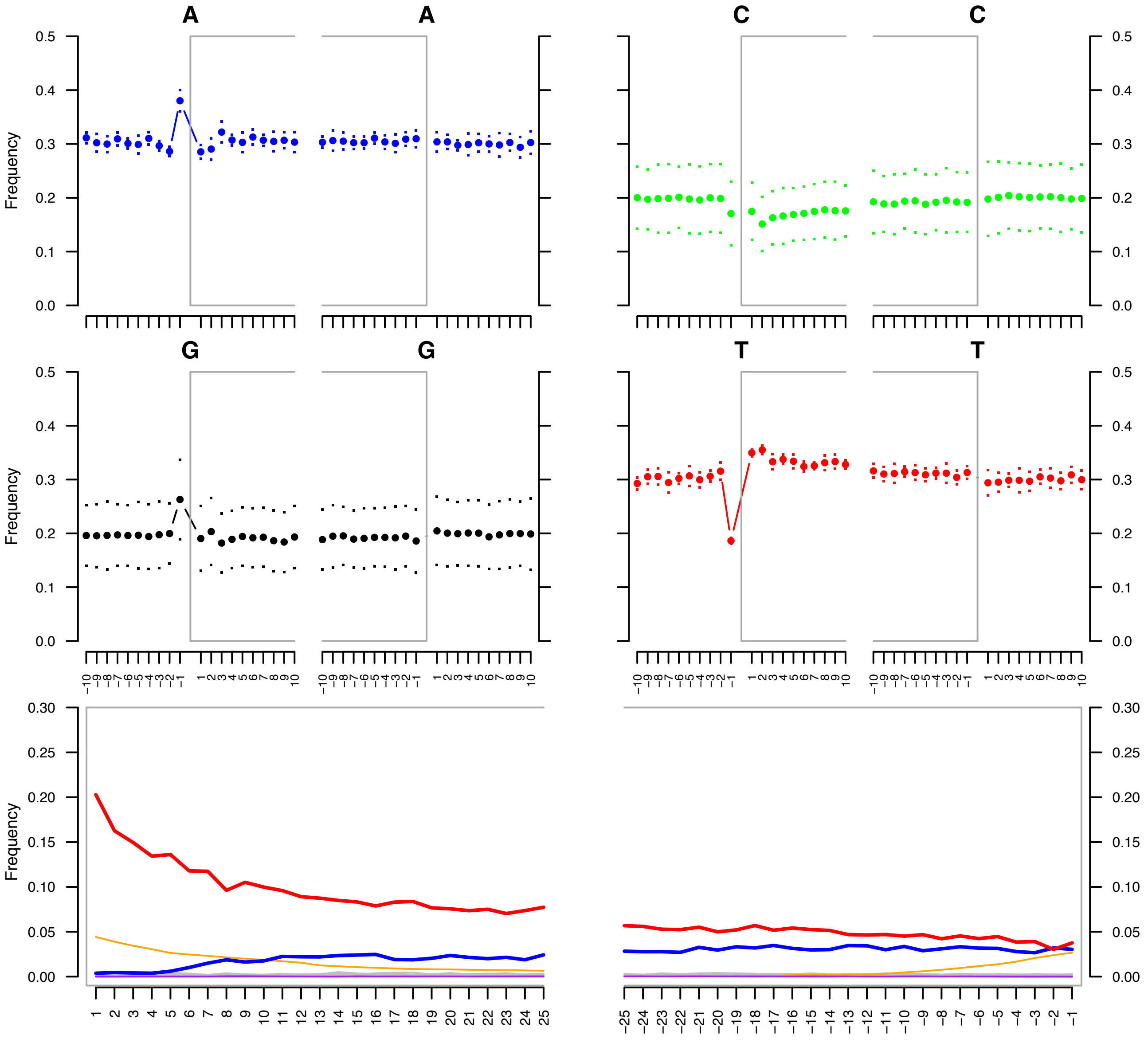

MS10136.merged

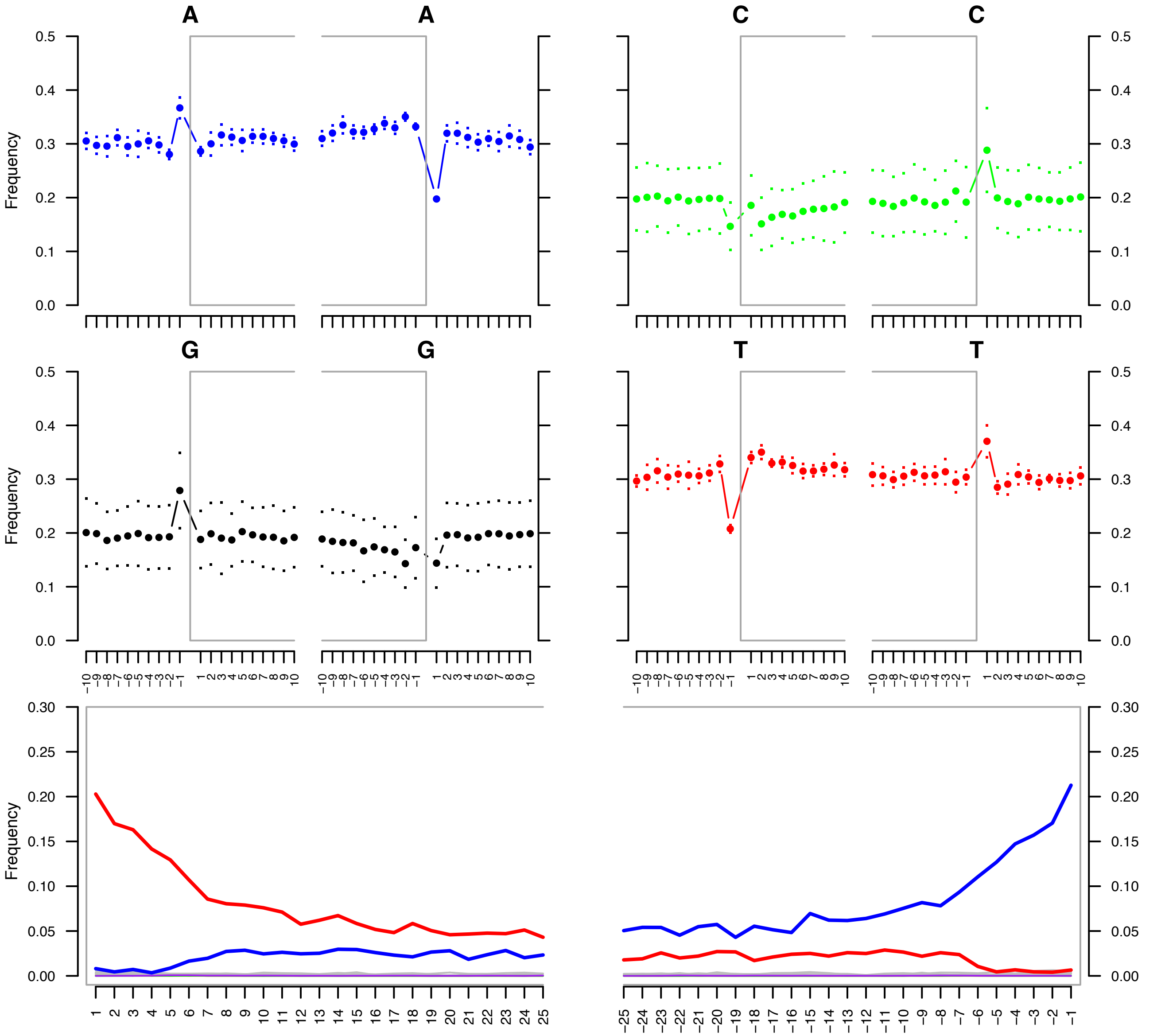

MS10137.unmerged

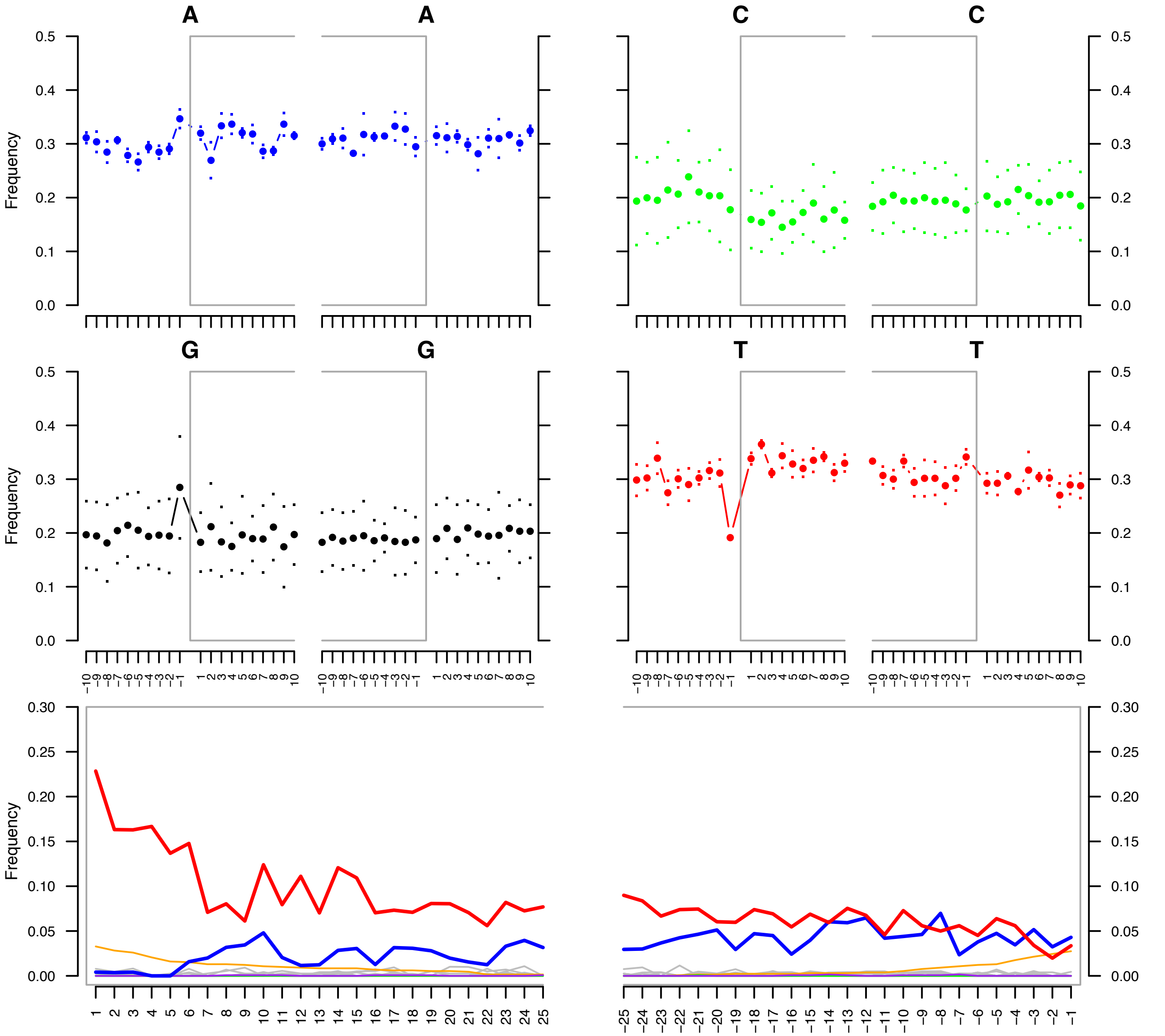

MS10137.merged

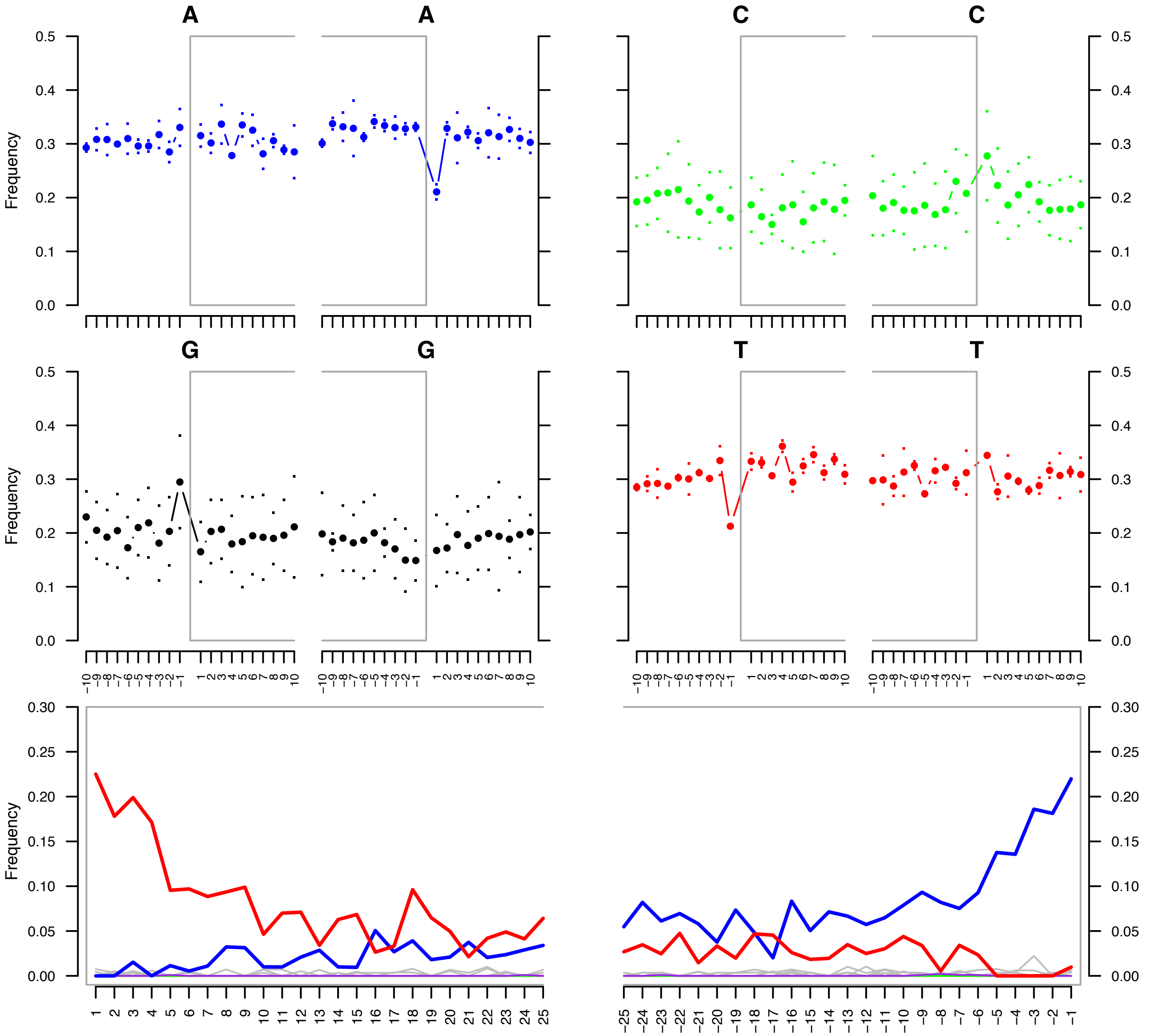

Supplement: S1 File — Top plots: Base frequency 5′ and 3′ of strand breaks. The gray brackets indicate start and end of molecules (strand breaks). Purines (A and G) show an elevated frequency before strand breaks. Bottom plots: C to T and G to A nucleotide misincorporations at the first and last 25 bases of endogeneous mtDNA fragments from the Wairau Bar dog sample [46]. (PDF) [file pone.0138536.s004.pdf]
