# Supplementary material for: Complete Mitochondrial Genomes of New Zealand’s First Dogs
Source: PLoS One. 2015 Oct 7;10(10):e0138536. doi: 10.1371/journal.pone.0138536 (PMC4596854; doi:10.1371/journal.pone.0138536)
Supplement: S1 Table — (DOCX) [file pone.0138536.s005.docx]

| Wairau Bar Dog Specimen No. | Otago Archaeological Laboratories Database Details | GenBank Accession No. | Coverage (%) | Mean read depth | Standard deviation of read depth | Imputed variable sites (%) | Used in final analysis |
| --- | --- | --- | --- | --- | --- | --- | --- |
| MS10062 | WB-CSA-30; BQ-5 | KT168373 | 99.928 | 94.11221 | 30.62578 | 0.01887 | y |
| MS10064 | WB-CSA-65; BQ-12 | n/a | 45.328 | 0.76284 | 1.09689 | * not used | n |
| MS10065 | WB-CSA-56; BQ-2 | KT168371 | 100.000 | 189.50487 | 28.39455 | 0 | y |
| MS10066 | WB-CSA-84; BQ-1 | KT168372 | 99.647 | 66.28379 | 23.89453 | 0.01887 | y |
| MS10067 | WB-CSA-84; BQ-2 | n/a | 78.311 | 1.86035 | 1.62678 | * not used | n |
| MS10068 | WB-CSA-56; BQ-1 | KT168369 | 99.857 | 27.68781 | 9.32758 | 0.03774 | y |
| MS10069 | WB-CSA-68; BQ-7 | KT168370 | 100.000 | 424.91863 | 80.98381 | 0 | y |
| MS10070 | WB-CSA-68; BQ-7 | KT168374 | 99.988 | 239.79584 | 64.56409 | 0 | y |
| MS10129 | WB-C-27 | KT168375 | 98.655 | 11.61177 | 5.59621 | 0.13208 | y |
| MS10130 | WB-C-27 | KT168378 | 99.002 | 14.86602 | 6.95936 | 0.09434 | y |
| MS10131 | WB-C-27 BQ | KT168379 | 99.707 | 46.41711 | 11.28736 | 0.01887 | y |
| MS10132 | WB-C-85 BQ | KT168376 | 99.713 | 21.39953 | 6.92298 | 0.03774 | y |
| MS10133 | WB-C-73 | KT168377 | 99.976 | 46.25426 | 12.09964 | 0.01887 | y |
| MS10135 | WB-C-142 BQ | KT168382 | 99.88 | 37.31058 | 9.70366 | 0.03774 | y |
| MS10136 | WB-D-31 BQ | KT168380 | 100.000 | 206.85479 | 42.26062 | 0.01887 | y |
| MS10137 | WB-D-6 BQ | KT168381 | 99.229 | 10.89687 | 4.46003 | 0.07547 | y |
