# Supplementary material for: Complete Mitochondrial Genomes of New Zealand’s First Dogs
Source: PLoS One. 2015 Oct 7;10(10):e0138536. doi: 10.1371/journal.pone.0138536 (PMC4596854; doi:10.1371/journal.pone.0138536)
Supplement: S2 Table — (DOCX) [file pone.0138536.s006.docx]

| Wairau Bar Dog ID No. | Human (NC_012920.1) | Chicken (NC_001323.1) | Cow (NC_006853.1) | Pig (NC_012095.1) |
| --- | --- | --- | --- | --- |
| MS10062 | 0.0 | 0.0 | 0.000356071010733 | 5.08672872476e-05 |
| MS10065 | 0.00410667207463 | 0.0 | 0.00258568241736 | 2.53498276212e-05 |
| MS10066 | 7.75735008921e-05 | 0.0 | 0.0 | 0.0 |
| MS10068 | 0.000342583076396 | 0.0 | 0.000171291538198 | 0.0 |
| MS10069 | 4.76043129508e-05 | 0.0 | 0.00174945850094 | 2.38021564754e-05 |
| MS10070 | 0.0 | 0.0 | 0.00122447194647 | 2.18655704727e-05 |
| MS10129 | 0.000414765657404 | 0.0 | 0.000414765657404 | 0.0 |
| MS10130 | 0.00480923372876 | 0.0 | 0.000320615581917 | 0.0 |
| MS10131 | 0.000300751879699 | 0.0 | 0.00220551378446 | 0.0 |
| MS10132 | 0.00108014689998 | 0.0 | 0.000216029379996 | 0.0 |
| MS10133 | 0.00129844186976 | 0.0 | 0.0061925689173 | 0.0 |
| MS10135 | 0.00038333759264 | 0.0 | 0.00332225913621 | 0.0 |
| MS10136 | 0.000481894533939 | 0.0 | 0.00117031529671 | 2.2947358759e-05 |
| MS10137 | 0.0075055187638 | 0.0 | 0.0 | 0.0 |
