# Supplementary material for: Complete Mitochondrial Genomes of New Zealand’s First Dogs
Source: PLoS One. 2015 Oct 7;10(10):e0138536. doi: 10.1371/journal.pone.0138536 (PMC4596854; doi:10.1371/journal.pone.0138536)
Supplement: S3 Table — (DOCX) [file pone.0138536.s007.docx]

|  | 15630 | 15642 | 15648 | 15655 |
| --- | --- | --- | --- | --- |
| Dog mitochondrial reference sequence (NC_002008) | A | T | A | G |
| Arc1 | G | A | A | G |
| Arc2 | G | A | G | A |
| MS10062 | G | A | G | A |
| MS10064 | G | A | G | A |
| MS10065 | G | A | G | A |
| MS10066 | G | A | G | A |
| MS10068 | G | A | G | A |
| MS10069 | G | A | G | A |
| MS10070 | G | A | G | A |
| MS10129 | G | A | G | A |
| MS10130 | G | A | G | A |
| MS10131 | G | A | G | A |
| MS10132 | G | A | G | A |
| MS10133 | G | A | G | A |
| MS10135 | G | A | G | A |
| MS10136 | G | A | G | A |
